# Genome-Wide Transcriptional Profiling to Elucidate Key Candidates Involved in Bud Burst and Rattling Growth in a Subtropical Bamboo (*Dendrocalamus hamiltonii*)

**DOI:** 10.3389/fpls.2016.02038

**Published:** 2017-01-11

**Authors:** Abhishek Bhandawat, Gagandeep Singh, Romit Seth, Pradeep Singh, Ram K. Sharma

**Affiliations:** ^1^Molecular Genetics and Genomics Lab, Department of Biotechnology, CSIR-Institute of Himalayan Bioresource TechnologyPalampur, India; ^2^Department of Biotechnology, Panjab UniversityChandigarh, India

**Keywords:** bamboo, cell cycle, environmental signaling, growth, RNA-Seq, spatio-temporal, transcriptome

## Abstract

Bamboo, one of the fastest growing plants, can be a promising model system to understand growth. The study provides an insight into the complex interplay between environmental signaling and cellular machineries governing initiation and persistence of growth in a subtropical bamboo (*Dendrocalamus hamiltonii*). Phenological and spatio-temporal transcriptome analysis of rhizome and shoot during the major vegetative developmental transitions of *D. hamiltonii* was performed to dissect factors governing growth. Our work signifies the role of environmental cues, predominantly rainfall, decreasing day length, and high humidity for activating dormant bud to produce new shoot, possibly through complex molecular interactions among phosphatidylinositol, calcium signaling pathways, phytohormones, circadian rhythm, and humidity responses. We found the coordinated regulation of auxin, cytokinin, brassinosteroid signaling and cell cycle modulators; facilitating cell proliferation, cell expansion, and cell wall biogenesis supporting persistent growth of emerging shoot. Putative master regulators among these candidates were identified using predetermined *Arabidopsis thaliana* protein-protein interaction network. We got clues that the growth signaling begins far back in rhizome even before it emerges out as new shoot. Putative growth candidates identified in our study can serve in devising strategies to engineer bamboos and timber trees with enhanced growth and biomass potentials.

## Introduction

With the growing human population, demand for food, shelter, land, and fuel has led to rapid loss of forest resources. These natural resources are largely abundant with slow growing timber species which require decades to attain full maturity. To overcome the rising demands associated with the exploding human population, there is a burning need for finding an efficient way to long term sustainability of forest resources. Bamboos (family: Poaceae) are fast growing, biomass abundant plants possessing tremendous ability to regenerate and produce plurality of growing shoots each year (Lessard and Chouinard, 1980). They attain full maturity in a short period of 3–8 years and thus can be potential sustainable bioresources (Chang and Wu, 2000). Unique physiochemical properties have made them commercially important for multiple applications including paper making, construction, handicraft, and food industries (Shukla and Das, 1981; Tewari, 1992; Das and Rout, 1994; Stevens, 1995). Furthermore, higher carbon fixation and oxygen emission rates compared to other trees makes bamboo a promising bioresource for carbon sequestration and climate change management (http://www.bamboocentral.org/shareinrepair/faq.htm). With these characteristics, bamboos have been fascinating plants among researchers since long. To explore their remarkable growth characteristics, comparative histological studies, monoclonal antibody bank creation, biochemical, and proteomic profiling of various developmental stages of growing shoot of bamboos have been done (Lee and Chin, 1960; Zheng et al., 1998; Lin et al., 2002; Li et al., 2007; Wang et al., 2011; Cui et al., 2012). Candidate genes (*Sucrose synthase, Cellulose synthase, BoSUT2, BoPAL1, Invertase*) associated with growth have been characterized in *Bambusa oldhamii* (tropical bamboo; Chiu et al., 2006; Hsieh et al., 2006, 2011; Chen et al., 2010; Gao et al., 2010). Past genome-wide efforts made to understand shoot growth were focused but limited to temperate bamboo (*P. edulis*) (Wang et al., 2009; He et al., 2013; Peng et al., 2013a,b). Moreover, India has 2nd richest bamboo bioresource after China, with ~130 species representing 18 genera (Kumar, 2004), spanning large area of temperate, subtropics and tropics. Previously, genetic marker based phylogeny clearly distinguished temperate and tropical/subtropical species (Sharma et al., 2008; Bhandawat et al., 2014, 2016; Bhandawat, 2016). *Dendrocalamus hamiltonii* (hexaploid; 2n = 6x = 72) is a giant, sympodial, fast growing subtropical bamboo with a life cycle of about 30 years and high commercial importance (Tewari, 1992; Bedell, 2006). *D. hamiltonii* has distinct environmental preferences compared to *P. edulis* to commence growth. New shoots emerge and attain maximum height (up to 16 m) within a single growing period of about 3–4 months in monsoon (July–October), and the shoot elongation ceases afterwards. Despite of wider distribution and adaptability of bamboos in subtropics and tropics (Li and Kobayashi, 2004), unique growth preferences, the decisive genome-wide efforts to understand various aspects of growth in subtropical bamboos remain elusive. In the present study, transcriptome of major vegetative developmental transitions of a subtropical bamboo, *D. hamiltonii* (*Dh*) was performed to determine the molecular basis of initiation and persistence of shoot growth. Additionally, efforts were also made to determine the molecular affinities and differences in the growth mechanisms of subtropical bamboo with previous studies on temperate species. Putative molecular clues identified in the study could help in devising strategies for widening cultivation boundaries, increasing the growth rate, and biomass potential of other slow growing bamboos.

## Materials and methods

### Phenological studies of shoot growth

**Figure 1:**
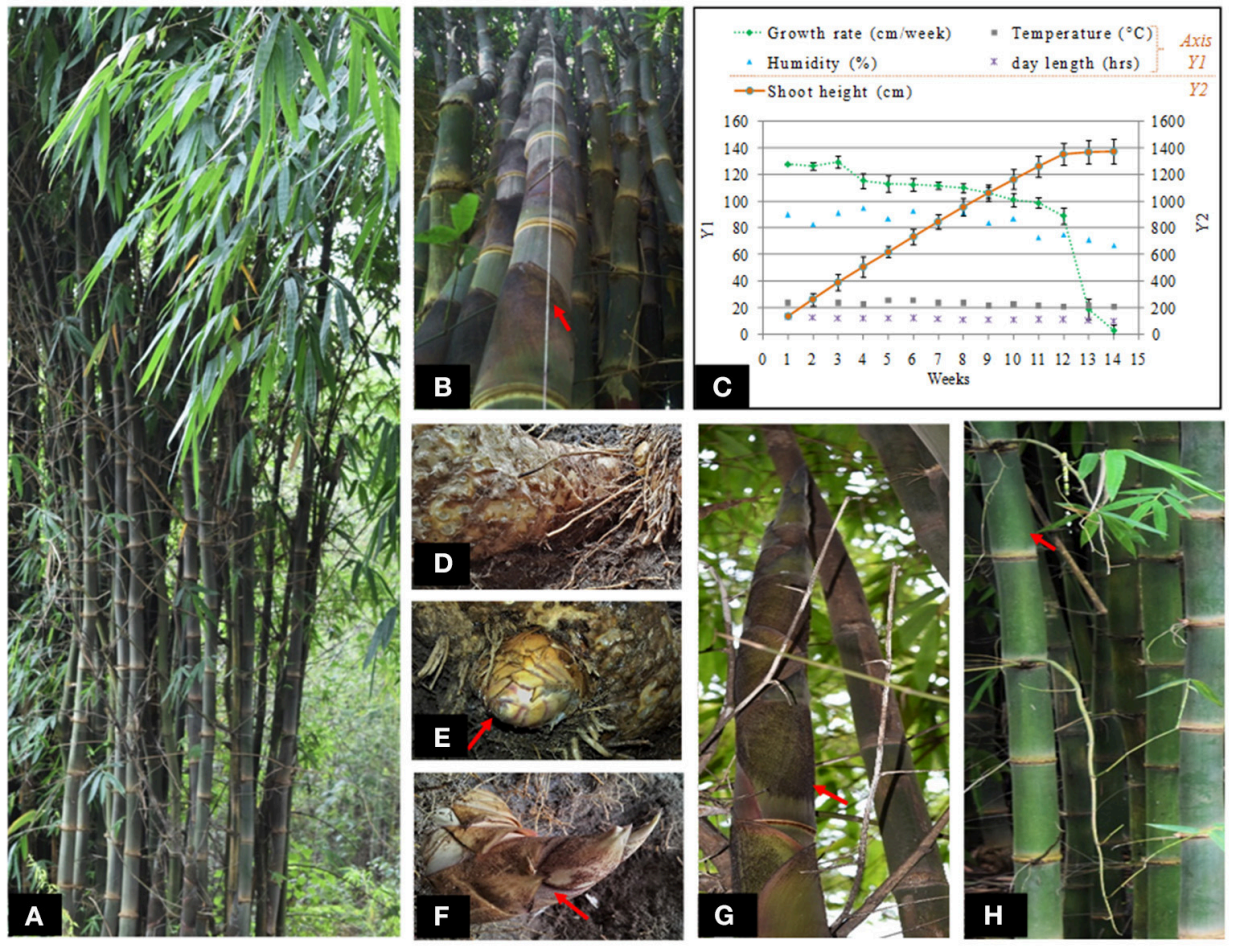
**Phenological observations and sampling strategies: (A)** Targeted *D. hamiltonii* clump, **(B)** growth rate estimation using “hanging-thread” method, **(C)** graphical representation showing correlation between shoot growth and environmental factors during monsoon, **(D–H)** samples procured for RNA-seq analysis: **(D)** dormant rhizome, **(E)** growing rhizome (bud), **(F)** growing shoot before internode development, **(G)** growing shoot after internode development, **(H)** mature shoot (after cessation of growth).

Phenological studies were performed to determine the growth characteristics of cultivated *D. hamiltonii (Dh)* maintained in natural condition at CSIR-Institute of Himalayan Bioresource Technology (CSIR_IHBT), India (32°6′N, 76°33′E; at an elevation of 1139 m). Three emerging culms (10 cm each) of similar physiological age were chosen to study the growth dynamics during monsoons. For quantifying the height of elongating shoots, a cheap and precise “hanging thread” method was used (Figure 1B). In this method, one end of the thread was gently tied at the tip of emerging shoot; the other end was left free and marked for measurement. Each morning (10 a.m.), shoot elevation was measured by relative shift of marker point; giving precise estimation of the growth rate. Correlation between growth rate and various environmental factors was estimated using Pearson correlation coefficient at *P* < 0.05.

### Sampling, cDNA library preparation, and sequencing

**Figure 2:**
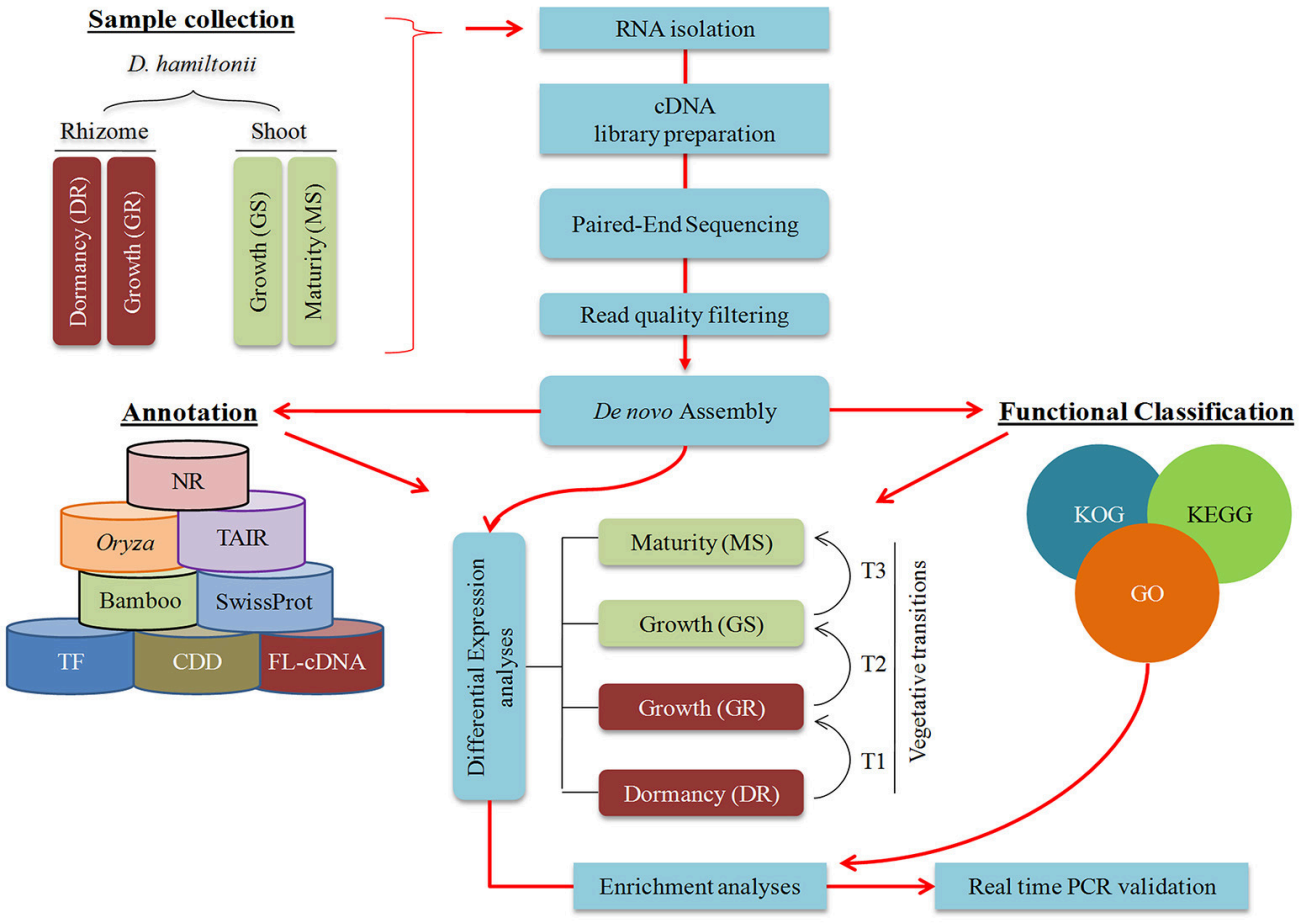
**Schematic representation of the approach used to dissect growth in ***D. hamltonii*****.

Rhizome and shoot samples of cultivated *Dh* were collected (Figures 1D–H) in the forenoon during winter dormancy (December) and monsoon (August–October) based on previous inferences (Zhang et al., 1995; Peng et al., 2013a) to obtain comprehensive overview of genes involved in growth. Dormant rhizome (DR) samples were procured during winters (December) when the day length was 10 h: 20 min and temperature ~9°C. As the growth is facilitated initially by cell division followed by cell expansion, growing shoot (GS) and growing rhizome (GR) were harvested twice; (i) before inter node development (etiolated shoot), to capture proliferating cells and (ii) shoot after attaining 3 m height (with distinct inter node) to capture cell expansion during monsoon (day length 13 h: 26 min to 12 h: 54 min; temperature 21–28°C). Mature shoot (MS) internode was harvested after attaining the maximum height with no observable shoot elongation up to 7 subsequent days (day length 10 h: 56 min; temperature ~19°C). All the samples were collected in triplicates from different culms, snap frozen in liquid nitrogen and stored at −80°C till further use. Schematic representation of the approach used in the current study is represented in Figure 2.

Total RNA was isolated from individual sample using IRIS protocol (Ghawana et al., 2011). The quantity was measured on Nanodrop (thermoscientific) and quality was assessed on 1% formaldehyde agarose gel. Triplicate RNA samples (3 each of DR, GR, GS, and MS) were pooled in equimolar concentration to prepare four samples for sequencing. Four micrograms of pooled RNA sample was used as input material for cDNA library preparation using Illumina Truseq™ RNA library preparation kit (Illumina Inc., CA, USA). cDNA libraries were quantified using Qubit 2.0 fluorometer (Invitrogen, USA) and their quality was assessed on Agilent 2100 Bioanalyzer (Agilent technologies, USA). For cluster generation, equimolar concentration (12 p.m.) of these libraries were loaded on sequencing flowcell. Sequencing of cDNA libraries was done using Illumina Genome Analyzer IIx (San Diego, CA) to generate 72 bp long paired-end (PE) reads. Along with increasing the depth of sequencing, paired-end sequencing improves the efficiency of *de novo* assembly (Shi et al., 2011; Liang et al., 2013).

### Transcriptome assembly

Fastq PE sequence data was generated using CASAVA package (Illumina). NGS QC Toolkit was used to filter out poor quality reads and adapters (Patel and Jain, 2012). High quality filtered reads were used for *de novo* transcriptome assembly using CLC Genomics Workbench (CLC Bio, Denmark) with default parameters (mismatch cost = 2; insertion cost = 3; deletion cost = 3; length fraction = 0.5; similarity fraction = 0.8) and sequence length cut-off = 300 bp.

### Functional annotation

Assembled transcripts were searched for putative function based on the sequence homology with publicly available protein databases, including *Phyllostachys edulis*, NCBI's NR, TAIR10, *Oryza sativa*, SwissProt, and plant transcription factor database (http://plntfdb.bio.uni-potsdam.de/v3.0/) using blastx with cut-off *E* = 1.0 e^−5^ (Pérez-Rodríguez et al., 2009). GO classification is a way of unifying functional characteristics of all biological systems. Based on GO classification (http://www.geneontology.org), assembled transcripts were classified into three categories, namely biological process, molecular function, and cellular component and visualized using WEGO software (Ye et al., 2006). Orthologs, the sequences conserved in different organisms were identified for the transcripts using eukaryotic cluster of orthologous groups (KOGs; Tatusov et al., 2003). KEGG (Kyoto Encyclopedia of Genes and Genomes) provides information about genes and pathways of various biological processes of an organism. KEGG Mapper v2.5 (http://www.kegg.jp/kegg/tool/annotate_sequence.html) was used to assign the KO terms and genes mapping to various metabolic pathways were determined. Transcripts which did not match any of the public databases were searched against NCBI's conserved domain database (http://www.ncbi.nlm.nih.gov/Structure/bwrpsb/bwrpsb.cgi) with *E*-value threshold of 1.0 e^−5^ (Marchler-Bauer et al., 2011).

### Identification of putative full length cDNAs

A high stringency criterion was used to identify putative full length cDNAs (FL-cDNAs) from assembled sequences. Firstly, blastx search (*E*-value 1 e^−10^, % identity ≥80) was conducted against SwissProt database; second, longest ORF was predicted using getorf (http://emboss.bioinformatics.nl/cgi-bin/emboss/getorf). Sequences with either, start (ATG) and stop codon (TAA/TAG/TGA), or start codon and hit with known protein homolog, were chosen as putative FL-cDNA.

### Read mapping and gene expression analysis

Reads from the individual sample were mapped to reference assembly using Bowtie2 v.2.2.4 and normalized to Reads Per Kilobase of transcript per Million mapped reads (RPKM) to measure the transcript abundance (Mortazavi et al., 2008; Langmead and Salzberg, 2012). Differentially expressed genes during three major transitions, namely T1 (DR to GR), T2 (GR to GS), and T3 (GS to MS) were determined using EdgeR package (Robinson et al., 2010). EdgeR identifies statistically significant and differentially expressed transcripts by comparing the read count values between two samples. Transcripts showing log_2_ fold change ≥ 2 (*P* ≤ 0.05 and FDR ≤ 0.05) were considered significantly differentially expressed. Heatmap depicting expression profiles of transcripts involved in growth were generated using MeV package v.4.9.0.

### Gene ontology enrichment

Differentially expressed transcripts were selected for GO enrichment analysis using singular enrichment analysis (SEA) of AgriGO (http://bioinfo.cau.edu.cn/agriGO). Rice orthologs (TIGR locus) were used against rice TIGR gene model as reference background. Plant GO slim was performed using Fischer statistical analysis with stringent Hochberg (FDR) adjustment value <0.01 for optimal enrichment of genes.

### Interactome analysis of key transcripts associated with bamboo growth

To further identify key genes involved in growth of bamboo, the growth related differentially expressed transcripts during T1 and T3 transitions including seven broad categories, namely environmental signal perception, epigenetic modulators, transcription factors, phytohormones, cell wall biogenesis, cell cycle regulators, and cell expansion were utilized for intractome analysis. Due to unavailability of significant protein information in bamboo, predetermined protein-protein interaction (PPI) network of *Arabidopsis thaliana* (ftp://ftp.arabidopsis.org/home/tair/Proteins/Protein_interaction_data/Interactome2.0/) was used for mapping the recognized transcripts as previously described (Jayaswall et al., 2016). Putative growth related transcripts were searched against Arabidopsis proteome to find putative targets using blastx (1e^−5^) and mapped to PPI network. Conserved correlation edge was determined on the basis of correlation between the growth related gene in bamboo supported by a significant correlation edge with its respective orthologs in the *A. thaliana* PPInetwork (AtPIN), using Cytoscape v3.4 (Smoot et al., 2011). First neighbor of targeted IDs was selected for predicting their interaction and to create the regulatory network. Based on the interactome statistics, putative master regulators were considered with their occurrence in hub based on their total interacting genes.

### Quantitative real-time PCR analysis

First strand cDNA was synthesized using 2 μg of total RNA of three random biological replicates each of DR, GR, GS and MS after DNase I treatment (Thermo Scientific, Lithuania, EU) using RevertAid™ H minus first strand cDNA synthesis kit (Thermo Scientific, Lithuania, EU) as per manufacturer's instructions. Gene specific qRT-PCR primers were designed using primer3 software (http://frodo.wi.mit.edu/; Table S1). qRT-PCR was performed in StepOne™ real-time PCR machine (Applied Biosystems, USA) using power SYBR Green qPCR master mix (Thermo Scientific, USA) following manufacturer's instructions. The conditions for qRT-PCR were kept as; 10 min at 95°C, 40 cycles each of 30 s at 95°C, 30 s at respective annealing temperatures and extension at 72°C for 30 s. The threshold cycles (C_t_) of individual target gene were averaged for triplicate reaction and normalized according to C_t_ of internal control (*cyclophilin*) as suggested earlier (Fan et al., 2013). This was followed with a melting curve program of 95°C for 1 min, annealing for 30 s, and 95°C for 30 s. Primer pairs showing a single melting temperature were used for analysis. The relative expression ratio of each gene was calculated using comparative C_t_ value method (Livak and Schmittgen, 2001). DR and MS were taken as control for estimating relative expression during T1 and T3 transitions, respectively. The fold change in expression was calculated and transformed to log_2_ scale.

## Results

### Phenology and growth dynamics

Based on morphological observations, we found bamboo initiates growth during monsoon characterized by abundant humidity and decreasing day length from 13 h:58 min in summer to about 13 h: 26 min. Growth (shoot elongation) continues throughout monsoon (day length 13 h: 26 min to 12 h: 54 min) and ceases once it attains maximum height (day length 10 h: 56 min). Phenological observations of growth (culm height and diameter) were carried out routinely during monsoon when the temperature ranged 21–28°C and humidity was 75–95%. New emerging shoots of height 10 cm were targeted for measurement to estimate the growth rate during monsoon (Figures 1A–C). We found that growth of bamboo spanned for about 14 weeks for attaining the maximum height (~14 m). Maximum average growth rate was found during initial weeks (127.7 cm/week). No visible growth was observed after 14th week as noted with sharp decline in growth rate. Moderate to high positive correlation of growth rate with humidity, temperature, and day length was measured (Table S2). Diameter at breast height (DBH) averaged 11.4 ± 0.6 cm after attaining the maximum height.

### Transcriptome sequencing

To obtain the comprehensive overview of genes involved in growth of bamboo, 4 vegetative stages, namely dormant rhizome (DR), growing rhizome (GR), growing shoot (GS), and mature shoot (MS), representing three major vegetative developmental transitions were sampled and subjected to paired-end transcriptome sequencing (Figures 1D–H). Over 66.5 million raw reads were obtained by sequencing various libraries. After adapter removal and quality filtering, 56.9 million clean reads (DR: 13.2 M, GR: 16.1 M, GS: 15.2 M, MS: 12.4 M) were obtained (Figure S1). As, reference for the species was unavailable, *de novo* assembly of high quality reads was performed. A total of 39,603 transcripts with an average length of 553 bp and N50 value of 559 bp were obtained (Table S3). However, for efficient mapping, assembly and precise reduction in number of genes, it is essential to develop a reference genome for the species (Vega-Arreguín et al., 2009). The raw reads of all the analyzed samples have been deposited to NCBI's Sequence Read Archive (SRA) with accessions SRR3822239 to SRR3822242 under BioProject, PRJNA328316.

### Transcript annotation

The functional annotation performed using blastx with NCBI NR (non-redundant), *O. sativa, P. edulis*, TAIR10, and SwissProt protein database annotated 73.3, 71.7, 70.4, 58, and 47.6% transcripts, respectively. *E*-value distribution of NR hits show that 65.1% of transcripts had strong matches (*E* ≤ 1e-45), while 34.9% of transcripts had moderate homology with *E*-value ranging between 1e-5 and 1e-45 (Figure S2). Based on blastx statistics (*E*-value, % query coverage, % alignment length, and bitscore values) *Dh* showed maximum similarity with *P. edulis* (temperate bamboo) proteins compared to other species.

Transcription factors act as molecular control of gene expression, regulating spatial, and temporal expression during various kinds of environmental responses (Nakashima et al., 2009; Wang and Wang, 2015). We identified 8976 transcription factor encoding transcripts belonging to 58 TF families, when searched against the plant transcription factor database. Among them, MYB family was most represented (684; 10.5%), followed by MYB related (468; 7.2%), and bHLH (451; 6.9%). Other important categories include WRKY (375; 5.7%), NAC (346; 5.3%), C2H2 (312; 4.8%), ARF (145; 2.2%), and E2F (62; 0.9%). Occurrence of top 15 transcription factor families is given in Figure S3. Summary of blastx searches against different protein databases is depicted in Figure S4.

Thirteen thousand six hundred and eighteen transcripts were assigned at least one functional term. In total, 50,139 GO terms were classified under three major categories, namely biological process (22 sub-categories), molecular function (13 sub-categories), and cellular component (10 sub-categories; Figure S5). Among biological processes, most assignments were given to cellular processes (GO:0009987; 37%), followed by metabolic processes (GO:0008152; 32.9%). Other important sub-categories include biological regulation (GO:0065007; 13.9%), response to stimulus (GO:0050896; 13.2%), developmental processes (GO:0032502; 8%), and growth (GO:0040007; 1.3%). Under molecular function, sequences were predominantly assigned to binding (GO:0005488; 35.2%) and catalytic activity (GO:0003824; 34.3%). Other sub-categories include transcription regulator activity (GO:0030528; 6.5%), transport activity (GO: 0005215; 5.4%), and molecular transducer activity (GO:0060089; 1.4%). Cell and cell part (GO: 0005623; GO:0044464) constituted a notable portion (49.2%) under cellular component category (25.9%).

**Figure 3:**
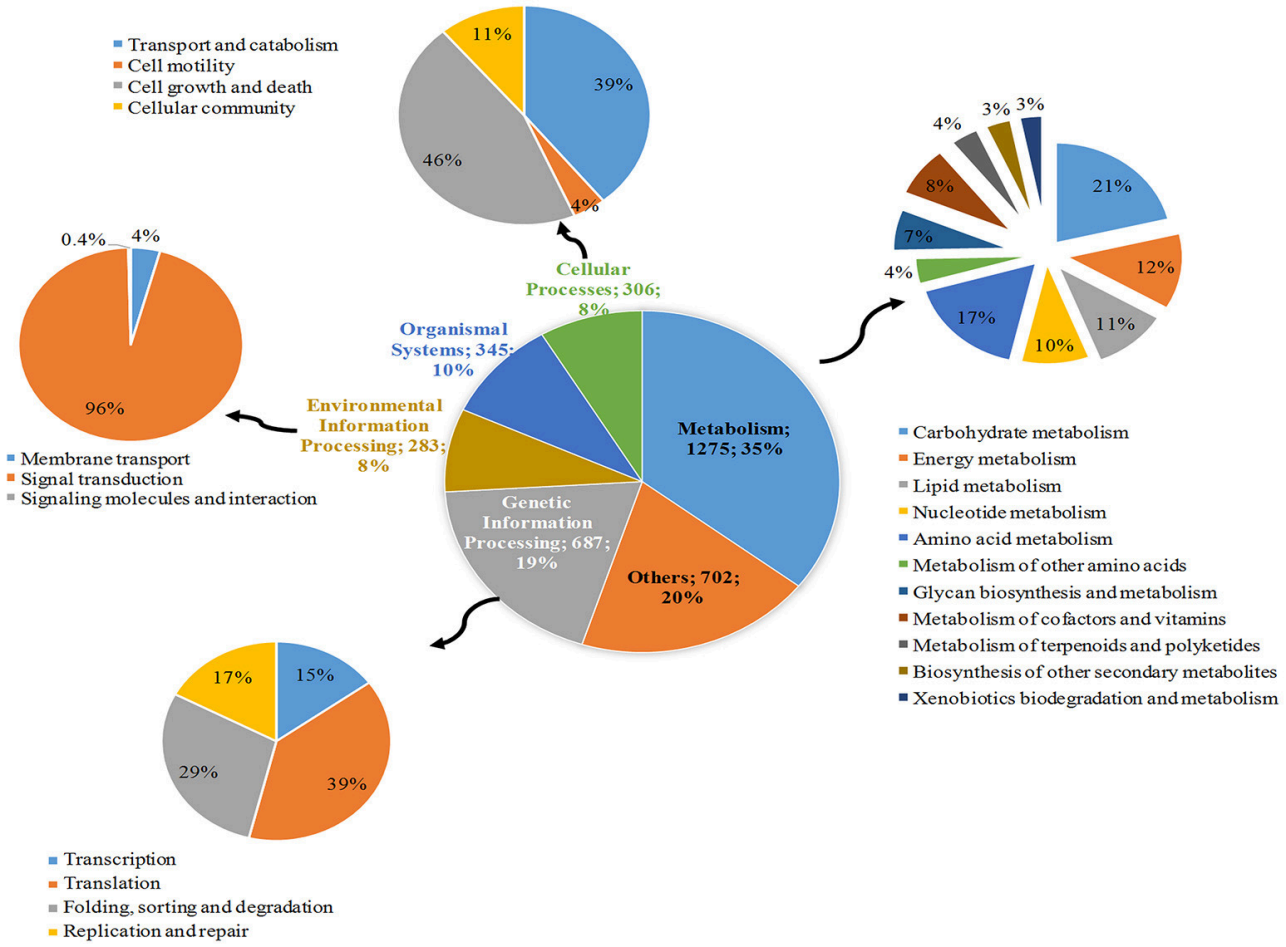
**KEGG pathways classification of ***Dh*** transcripts**.

To better establish links and assign a high-level function of genes in the genome, we performed KEGG analyses. Four thousand nine hundred and eighty three transcripts were assigned KO (KEGG Orthology) numbers in the KEGG classification system. Of these, 4799 were successfully mapped to 316 metabolic pathways, broadly classified under 6 categories (Figure 3). Maximum transcripts belonged to metabolism (35%) which includes carbohydrate metabolism (21%) and amino acid metabolism (17%). Other important sub-categories include glycan biosynthesis and metabolism (7%), which seems to support cell wall synthesis in developing shoot. Genetic information processing was the second major category (19%) which includes translation, (39%), protein folding and degradation (29%), replication and repair processes (17%), and transcription (15%). The transition from dormancy to growth occurs by sensing and transducing environmental cues in dormant rhizome. Under environment information processing (8%), signal transduction forms the major portion (96%) followed by membrane transport (4%) and signaling molecules and their interactions (0.4%). Cell growth and death (46%), and transport and catabolism of metabolites (39%) formed major fraction under cellular processes (8%).

**Figure 4:**
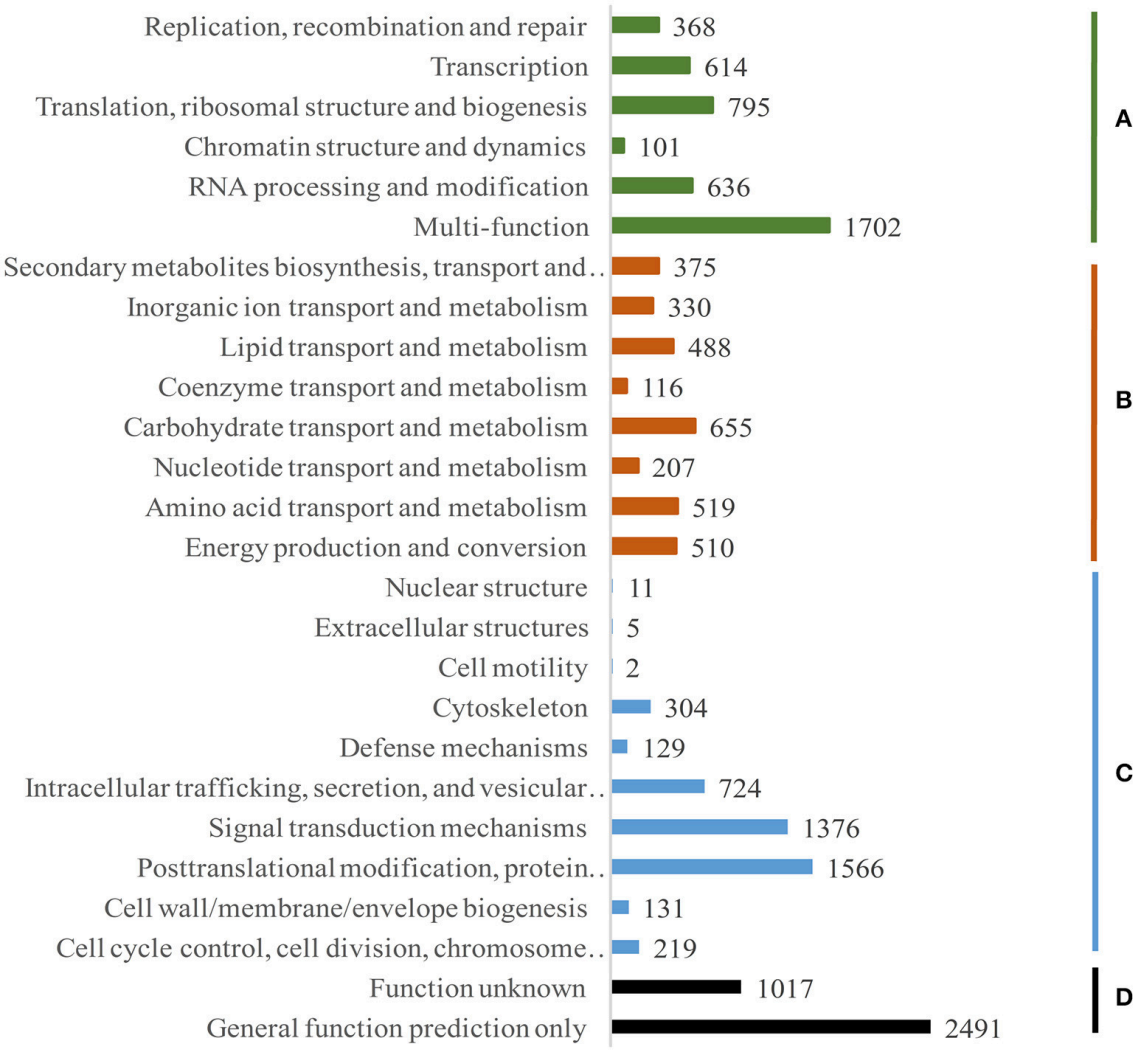
**Functional classification of ***Dh*** transcripts to cluster of Orthologous groups. (A)** Information storage and processing, **(B)** metabolism, **(C)** cellular processes and signaling, **(D)** poorly characterized.

For identification of evolutionary homologs (orthologous and paralogous) of proteins, we performed euKaryotic Conserved Orthologous Groups (KOG) analysis. Fifteen thousand three hundred and ninety one annotated transcripts were classified under 26 groups (Figure 4). It is worthy to note that important clusters include post translational modification, protein turnover, chaperones (1566 transcripts; 10.2%), signal transduction mechanisms (1376; 8.9%), carbohydrate transport and metabolism (655; 4.3%), energy production and conversion (510; 3.3%), cell cycle control, cell division, chromosome partitioning (219; 1.4%), and cell wall/membrane/envelope biogenesis (131; 0.9%). This indicates that these processes might play essential role in the rapid growth of bamboo. Apart from these, 11% (1702) were clustered under multiple function (MF) category. Apparently, a significant fraction (3508; 22.8%) was poorly characterized (D) and clustered under function unknown and general function categories.

In total, 30,240 transcripts (~76%) matched against at least one protein databases (*P. edulis*, NR, TAIR, Rice, SwissProt, TF) and/or categorized under KEGG, KOG, and GO classification. Conserved domains were identified in 21 transcripts that were not annotated to any of the searched databases. Based on ORF prediction and homology with SwissProt proteins, a total of 5198 putative full length cDNAs were predicted from current dataset (Table S4).

### Global changes in gene expression during dormancy and growth period

**Figure 5:**
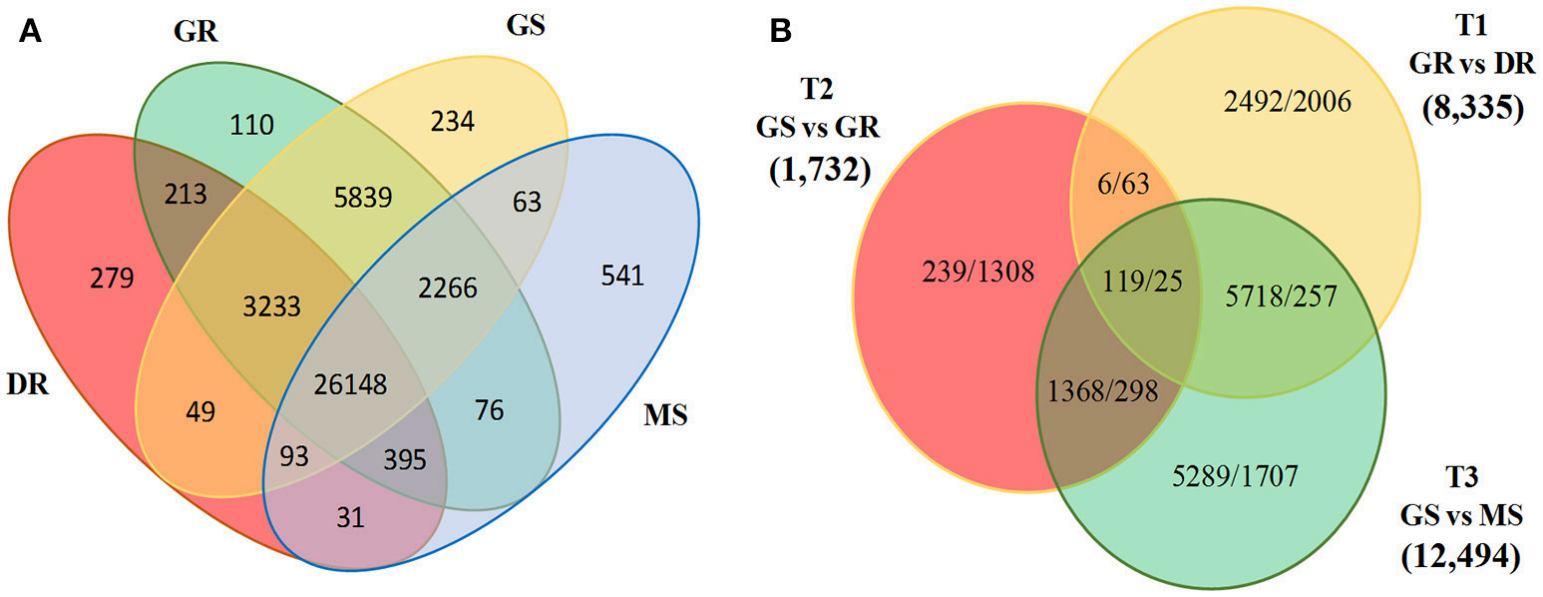
**Venn diagram representing (A)** phase specific abundance of transcripts commonly or uniquely expressed, **(B)** Abundance of differentially expressed transcripts (Up-regulated/down-regulated) under specific developmental transitions (DR, dormant rhizome; GR, growing rhizome; GS, growing shoot; MS, mature shoot).

Together with generating extensive genomic resource, RNA-Seq data provides an opportunity to study genome-wide expression of genes associated with trait of interest. To investigate differentially expressed transcripts during three vegetative transitions, namely T1, T2, and T3; we mapped individual sample reads to *D. hamiltonii* transcriptome assembly. A total of 28,210,924 high quality reads was mapped to the reference assembly. Transcript level of 19,798 (33.9%) genes changed significantly (log_2_ fold change ≥2) during one or more developmental transitions. In total, 26,148 transcripts were found to be commonly up-regulated in all the tissues. Huge number of transcripts were up-regulated in GS (12,494; 84.5%) and GR (8335; 78.0%) as compared to MS (2287; 15.5%) and DR (2351; 22%) during T3 and T1 transitions, respectively (Figure 5B). This might be due to the reason that both GS and GR comprise of actively growing cells and a large fraction of genes are likely to be transcriptionally active. Overall, maximum transcripts (14,781) were differentially expressed during T3 suggesting highest physiological dissimilarities between active shoot growth and maturity. This was followed by 10,686 transcripts differentially expressed during T1. Least number of transcripts (3426) was differentially expressed during T2 possibly due to active growth machinery, suggesting high physiological affinities between them (Figure 5B). Further, the differential expression of genes shows correlation between physiological state and tissue wise gene expression, with larger similarities between tissues (GS and GR, DR and MS) rather within tissues (GS and MS, DR and GR). Considering the maximum number of differentially expressed transcripts in T1 and T3 transitions, downstream analyses were performed emphasizing these two transitions only. Abundance of transcripts showing unique and overlapping expression during different developmental stages, and differentially expressed transcripts during major developmental transitions is summarized as a Venn diagram (Figures 5A,B).

### Gene ontology enrichment

To investigate the function of differentially expressed transcripts during various developmental transitions, stage wise GO enrichment analysis was performed (Table S5). Under biological processes, we found subcategories of developmental processes (embryonic development, post-embryonic development, anatomical structure morphogenesis), response to stimulus (response to stress, response to abiotic stimulus), cellular processes (cell growth, regulation of cell size, cell cycle), metabolic processes (primary metabolic processes including carbohydrate metabolism, DNA metabolic processes, protein modification processes), regulation of gene expression (epigenetic regulation) were up-regulated and highly enriched (FDR < 0.01), while subcategories under response to stimulus showed moderate to high enrichment in growing tissues (GS and GR; Figures S6, S7). Most of the sub-categories under cellular processes, metabolic processes, and regulation of gene expression were either not enriched or showed low enrichment in DR. Few categories like response to stimulus (response to stress and response to abiotic stimulus) were found highly enriched and up-regulated in the DR (Figure S8). This indicates that during dormancy the plant senses and responds to certain environmental stimulus. In mature shoot (MS), subcategories under metabolic processes and cellular processes (cellular biosynthetic process, generation of precursor metabolites, cellular macromolecular biosynthetic process, translation), response to stimulus (response to abiotic stimulus, response to stress) were most enriched suggesting that even mature shoot is physiologically active to maintain plant's vitality (Figure S9). The enrichment analysis suggests that GS and GR shares large fraction of enriched genes supporting rapid growth of bamboo culm during growth season.

### Identification of genes involved in fast growth of *D. hamiltonii*

Based on maximum number of differentially expressed transcripts during T1 and T3 transitions, 69 major gene categories (611 transcripts) found to be possibly involved in initiation and maintenance of growth of bamboo during monsoon were identified (Table S6). To represent overall growth these gene categories were grouped into 7 major categories: (A) Environmental signal perception, (B) Phytohormones, (C) Epigenetic modulators, (D) Transcription factors, (E) Cell cycle regulators, (F) Cell expansion, and (G) Cell wall biogenesis. Eight genes were found to be involved in sensing environmental stimulus and signal transduction pathways may possibly be involved in transition from dormancy to active growth (T1 transition). We found phosphatidylinositol (phosphatidylinositol 4-kinase, phosphoinositide phospholipase C, and protein kinase C) and calcium signaling pathway genes (Calmodulin) to be up-regulated in GR and GS during T1 and T3 transitions, respectively. Genes of MAP kinase family, anaphase promoting complexes (APCs), water transport proteins (aquaporins), and response to humidity (copine) were found to be up-regulated in actively growing tissues.

Phytohormones are among the major regulators of plant development. We found 14 genes involved in biosynthesis, binding, and transport of various hormones including auxin, cytokinin, and ethylene to be differentially expressed in T1 and T3 transitions. Receptors for cytokinin, gibberellin, and brassinosteroids were found up-regulated in growing tissues during T1 and T3 transitions.

Among various transcription factors, we found up-regulated expression of SCARECROW (GRAS family), E2F, Auxin response factor (ARF), WD40, AINTEGUMENTA (AP2/ERF), BES1 interacting TF (HLH) in growing tissues during T1 and T3 transitions. WUSCHEL homeobox (WOX) was also found up-regulated in the growing tissues. KNOTTED-1-like homeobox (KNAT) was found down-regulated in GS, while Growth Regulating Factor (GRF) was found up-regulated in GS during T3 transition.

Twelve cell cycle regulatory genes, including cyclins and CDKs were highly and differentially expressed in the growing phase of T1 and T3 transitions. We found genes for RNA polymerase II mediators, MCM and BIG BROTHER to be up-regulated in growing tissue (in T1 and T3 transition). Few genes for controlling cell proliferation like RBR, CLAVATA1, PASTICCINO, TEOSINTE BRANCHED, POM1, SKP1, and ERECTA were differentially expressed in T1 and T3 transitions.

**Figure 6:**
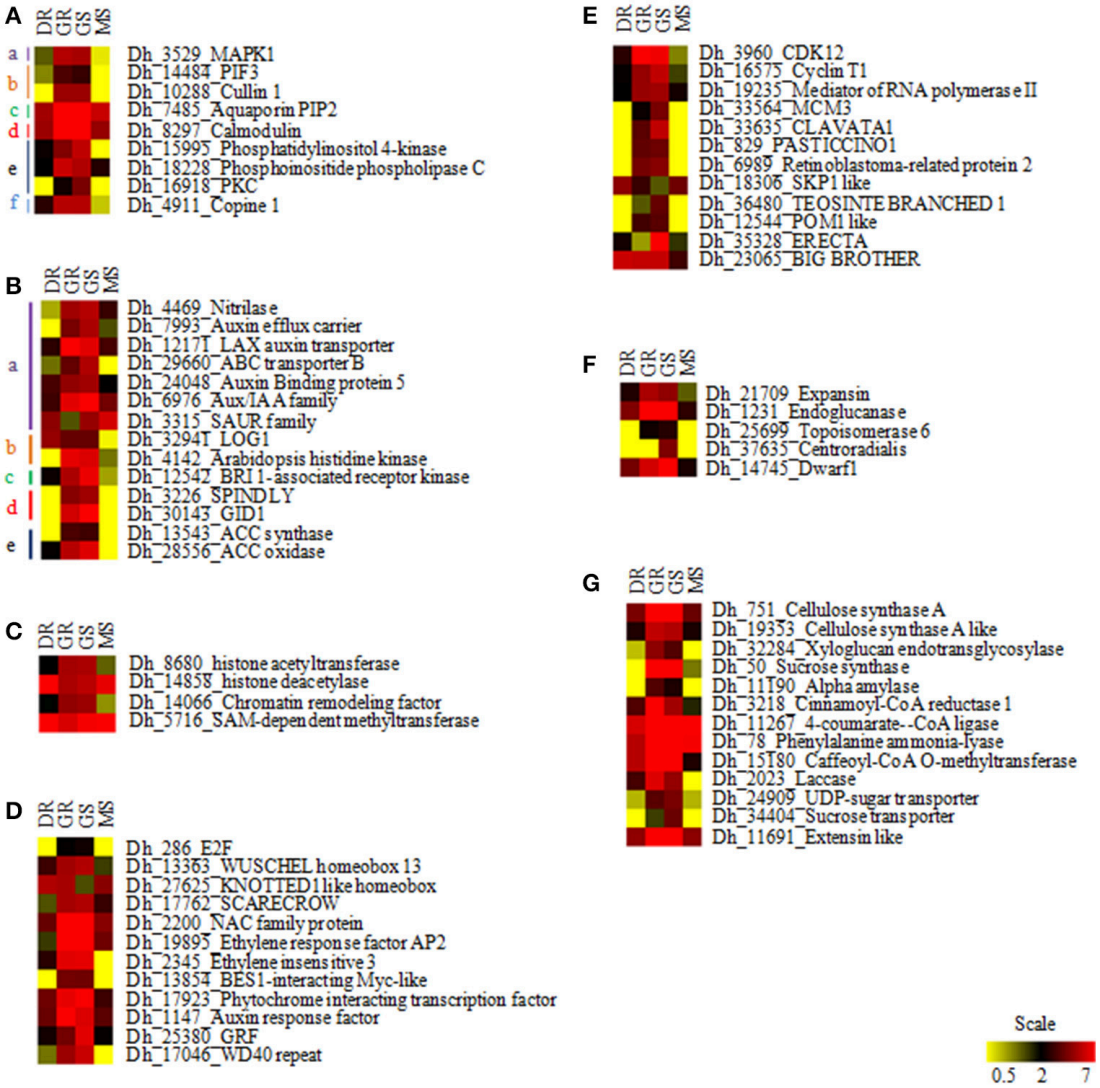
**Heatmap showing transcript abundance of growth-associated genes in different vegetative stages of ***D. hamiltonii*** generated using MeV**. Scale at the bottom represents relative expression. Red represents highly expressed yellow represents poorly expressed genes. **(A)** Environmental signal perception: a, mitogen associated; b, Circadian rhythm; c, water transport; d, calcium signaling; e, PI signaling; f, response to humidity, **(B)** Phytohormone related genes: a, auxin; b, cytokinin; c, brassinosteroid; d, gibberellin; e, ethylene, **(C)** Epigenetic modulators, **(D)** Transcription factors, **(E)** Cell cycle regulators, **(F)** Cell expansion, and **(G)** Cell wall biogenesis.

Eighteen genes involved in cell expansion and biogenesis of cell wall like expansin, endoglucanase, cellulose synthase, extensin, laccase were up-regulated in growing tissues (in T1 and T3 transitions). Genes providing building blocks and metabolic energy, including sucrose synthase, α-amylase, sucrose, and nucleotide sugar transporters were also found up-regulated in growing tissues during T1 and T3 transition. Lignin biosynthesis related genes like cinnamoyl-CoA reductase, 4-coumarate–CoA ligase, phenylalanine ammonia-lyase, and caffeoyl-CoA O-methyltransferase were also found differentially expressed in T1 and T3 transitions. Abundance of differentially expressed transcripts in 4 vegetative stages is represented as a Heatmap in Figure 6.

### Protein-protein interactions among genes involved in growth

**Figure 7:**
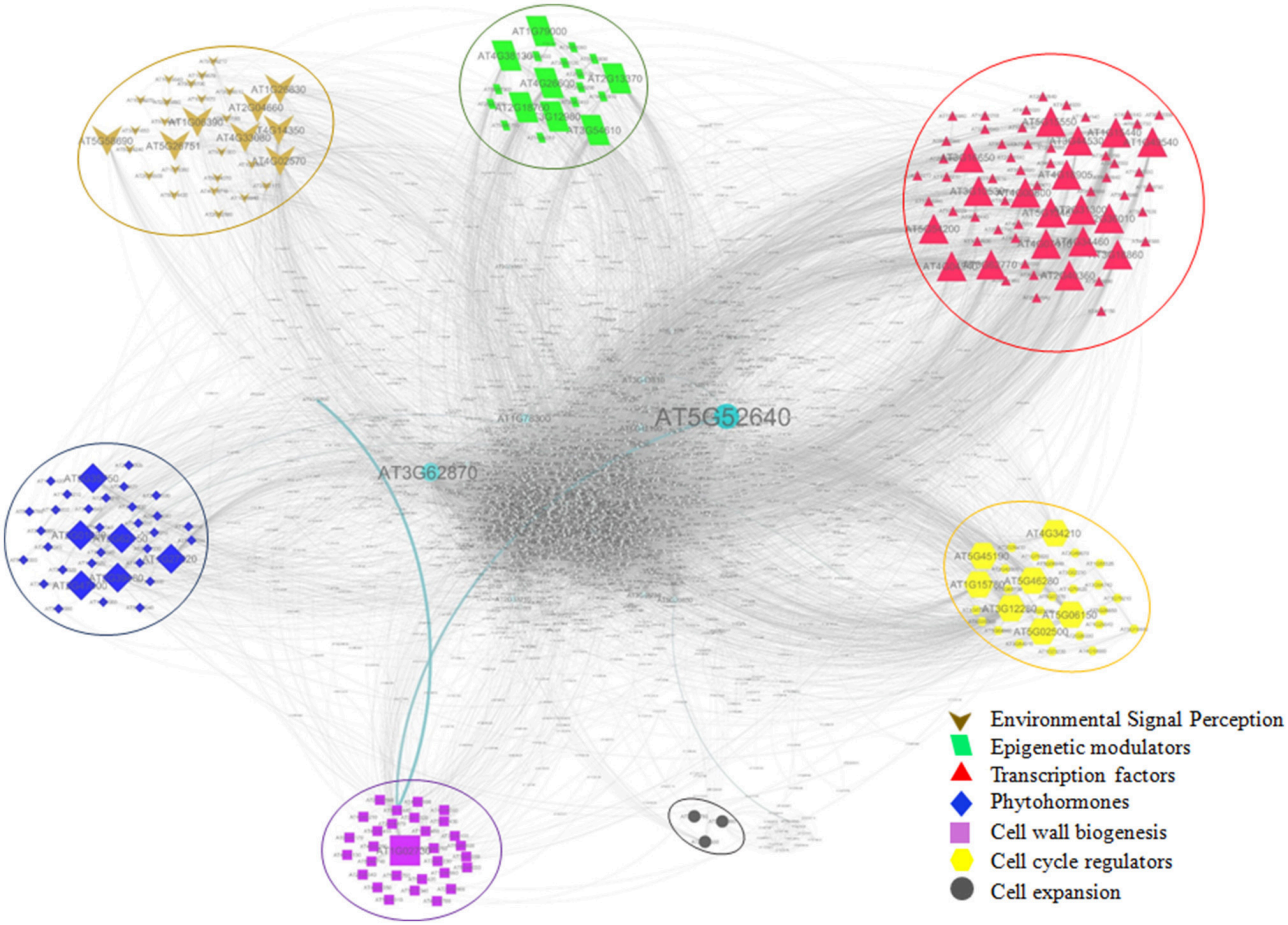
**Protein-protein interaction network (PPIN) of growth related genes**. Categories of growth related genes (Environmental signal perception; Epigenetic modulators; Transcription factors; Phytohormones; Cell wall biogenesis; Cell cycle regulators; Cell expansion) are depicted with enlarged symbols indicating key genes occurring in the major hubs.

For identification of key genes among the putative growth related genes involved in growth of bamboo, predetermined protein-protein interaction (PPI) network of Arabidopsis was used. Out of 611 growth related transcripts, 310 unique TAIR-IDs were successfully assigned based on blastx against Arabidopsis proteome. Among these, 213 belonging to various growth categories: Environmental signal perception (30), Phytohormones (35), Epigenetic modulators (19), Transcription factors (65), Cell cycle regulators (30) cell expansion (3) Cell wall biogenesis (31) were mapped, and found to be interacting with 2679 nodes with clustering coefficient 0.27. Average number of undirected neighbors in the network for each gene was found ~27. Critical analysis revealed 47 of the 213 mapped genes were found as the major hub containing 2019 nodes with average number of neighbors 30.854. Based on the interactome, 47 genes identified as the major hub can be considered as potential genes for the growth (Table S7). The PPI network analysis revealed that genes involved in perceiving the environmental signals were found to interact with 335 other genes, Phytohormones with 299, Epigenetic modulators with 456, Transcription factors with 965, Cell cycle regulators with 627 genes, and Cell wall biogenesis with 49 genes; therefore, can be potential candidates for growth dissection in bamboo (Figure 7).

### Validation of RNA-Seq expression data by qRT-PCR

**Figure 8:**
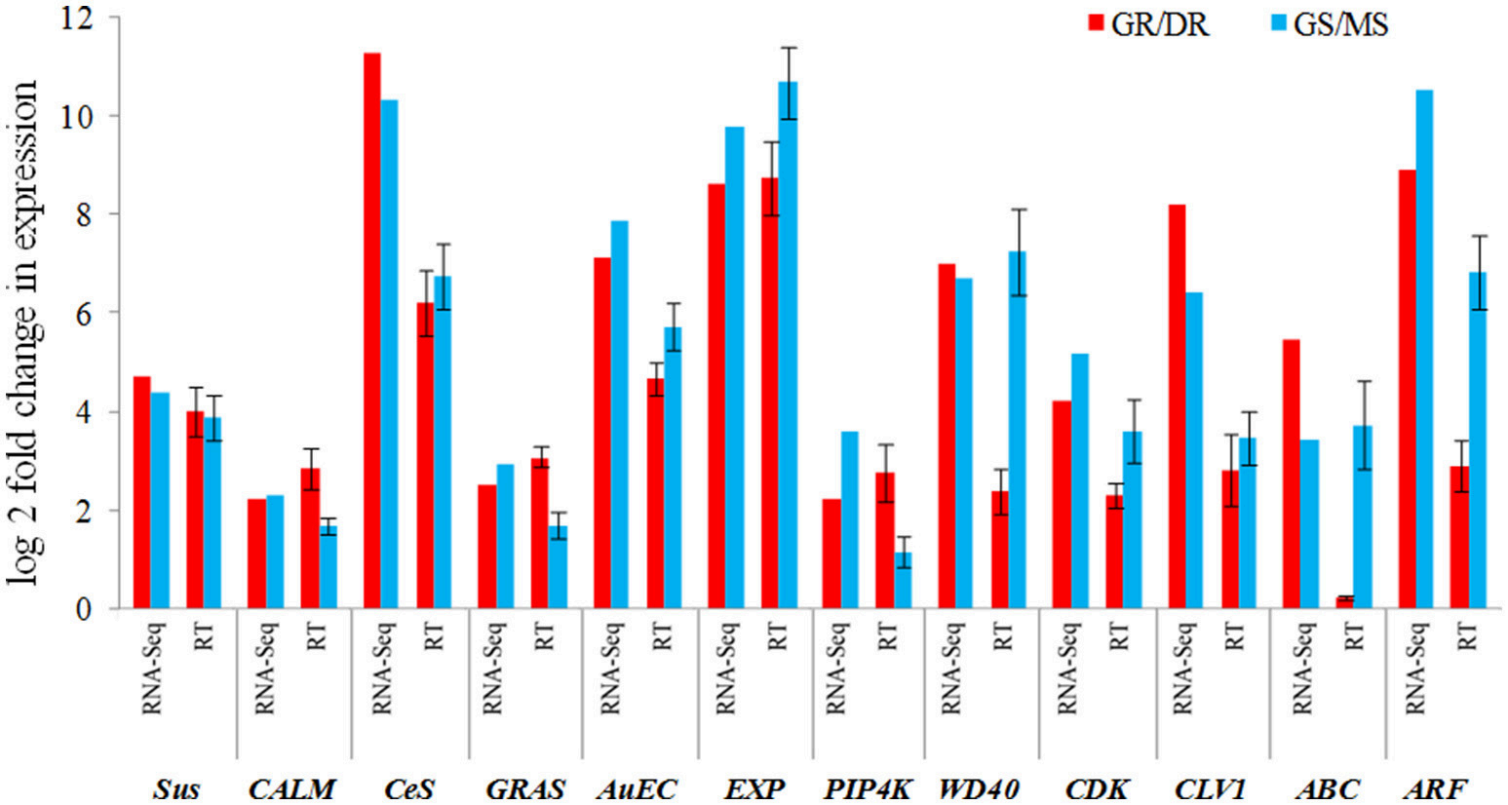
**Comparison between transcriptome and qRT–PCR expression profiles of 12 selected growth related genes (***Sus***, sucrose synthase; ***CALM***, calmodulin; ***Ces***, cellulose synthase; ***GRAS***, GRAS family TF; ***AuEC***, auxin efflux carrier; ***EXP***, expansin; ***PIP4K***, phosphatidylinositol-4-kinase; ***WD40***, WD40 repeat; ***CDK***, cyclin dependent kinase; ***CLV1***, clavata1; ***ABC***, ABC transcporter; ***ARF***, auxin response factor)**. Error bar represents the SD for replicated experiments.

To verify the reliability of RNA-Seq differential expression analyses, quantitative real time PCR was performed for 12 selected genes playing significant role in growth. Two genes, phosphatidylinositol 4-kinase (*PIP4K*) and calmodulin (*CALM*) belonging to environmental signal processing were found up-regulated in growing tissues during T1 and T3 transitions. However, the most significant up-regulation was seen in GR (T1) suggesting their role in bud burst (growth initiation). Three transcription factors, namely GRAS, WD40, and ARF family involved in meristem function were found up-regulated in growing tissues in both T1 and T3 transitions. CDK and CLV1 having a role in regulating the cell cycle was found to be up-regulated in GR and GS during T1 and T3 transitions. Two major auxin transporters, auxin efflux carrier and ABC proteins were up-regulated in the growing tissues. Cell wall elongation gene (Expansin: *EXP*) and cell wall biogenesis genes were found up-regulated in the growing tissues during T1 and T3 transition. Majority of the genes expressed similar pattern in both the transitions, T1 and T3 in qPCR analysis too (Figure 8).

## Discussion

Being one of the fastest growing plants, bamboo appears as a model system to understand growth. Phenological observation suggests that growth in *Dh* begins soon with the onset of monsoon and persists till the plant attains its maximum height. Growth dynamics reveals that the maximum growth occurs during early weeks of monsoons with subsequent declination in the growth rate, while shoot elongation finally ceases after attaining its maximum height. In contrast to *P. edulis* (temperate bamboo) which is a monopodial bamboo and grows in spring (April to May), *D. hamiltonii* (subtropical bamboo) is a sympodial bamboo which grows in monsoon (July–October). At one side *P. edulis* senses dry and increasing day length of spring, wherein *D. hamiltonii* multiple environmental cues like excessive rainfall, high moisture and decreasing day length appears to be driving forces to initiate bud burst in dormant bamboo rhizome based on our phenological observations. Moderate to strong positive correlation between environmental factors (humidity, temperature, and day length) and growth rate suggests their role in consistent growth of bamboo shoot. Rhizome provides an instant and continuous supply of stored sugar (starch) and is crucial to understand the genes involved in the initiation and persistence of growth during monsoon. We performed genome-wide molecular studies in subtropical bamboo *D. hamiltonii* (*Dh*) using spatio-temporal deep transcriptome sequencing of four vegetative phases, enriching the genomic resource for *Dh*. Total raw data generated in our study was comparable to that of recently published transcriptome of *Dendrocalamus sinicus* (Cui et al., 2016). We performed sequence comparison and classification with most of the available databases in order to acquire maximum functional information. Maximum transcripts showed similarity with three species of Poaceae (grass family) implying that the transcripts were assembled adequately. Number of blast hits with rice was slightly more than with *P. edulis* may be because rice has been extensively studied at gene and protein level; however, the bitscore values with *P. edulis* was significantly higher than rice homologs suggesting higher sequence conservation between the bamboo species. Number of transcripts matching with the public databases was almost same as that found in *D. sinicus*, possibly because both species belong to same genus. Substantial number of genes appeared specific to *D. hamiltonii* with no orthologs in other organisms as predicted based on the sequence homology, while a significant number of differentially expressed transcripts with unknown function, possibly play important role in growth of bamboo. Interestingly, a large number of metabolic pathways genes involved in growth were identified. Inclusion of rhizome under study resulted in significantly larger number of differentially expressed transcripts than reported earlier (Peng et al., 2013a). Maximum transcripts were found differentially expressed during T3 transition (GS to MS) suggesting highest physiological dissimilarities between growing and mature tissues. A set of 12 differentially expressed genes were further validated using qPCR. The expression pattern of most of the genes obtained through qPCR and RNA-seq analyses was in good agreement with each other. Few genes such as PIP4K, WD40, and ABC showed variable pattern in qPCR analysis, this may be because of difference in normalization procedures, wherein, differential expression with edgeR normalizes expression values in pair-wise comparison across all transcripts, while in qPCR each individual sample is normalized based upon its relative expression with respect to housekeeping gene. However, in both the cases, all the genes recorded up-regulated trend in growing tissues during both the transitions (T1 and T3) irrespective of their magnitude.

Through GO enrichment of differentially expressed genes, we observed high enrichment of response to stimulus category genes during unfavorable environmental conditions which might be essential for rhizome dormancy, whereas most of the metabolic processes were poorly enriched. With the onset of monsoon, the plant perceives certain environmental cues like rainfall, change in soil osmolarity, temperature, humidity, and changing day length activating dormant rhizome bud to commence growth. Growing rhizome (GR) transforms to emerge out as new shoot (GS) during early monsoons. Analyzing differential expression results in T1 transition, it seems that molecular processes involved in growth begins far back in rhizome before it emerges as new shoot. As evident from enrichment analysis of GR and GS, categories involved in response to stimulus, cellular and metabolic processes (morphogenesis, cell growth, and cell size regulation), epigenetic regulation of gene expression were highly enriched, might involved in overall growth of bamboo. Most of the carbon sequestration and biomass accumulation occurs before shoot matures, as- evident from enrichment of corresponding biological processes (morphogenesis, cell growth, primary metabolic processes) in our analyses in GR and GS (Yen, 2016). Once the shoot attains maximum height, processes involved in cell division and expansion stop leading to cessation of visible growth in bamboo culm. However, in mature shoot, primary cellular and biosynthetic processes, macromolecule synthesis continues to support plant's basic physiological processes.

The PPI network analysis helped us to identify growth related potential genes in bamboo. Based on the interactome, direct or indirect interaction between genes of environmental signal perception (PI4K, cullin, PIF3), cell cycle regulation (cyclin, RBR, E2F, SKP1, RNA polymerase II mediator) and phytohormones (brassinosteroid and cytokinin receptor), auxin and sugar transporters, and cellulose synthase were observed. A direct interaction of WD40 repeat containing transcription factors with epigenetic modulators (methyltransferases, histone acetylase, and histone deacetylase), cell cycle regulators (mediator of RNA polymerase II), environmental signaling (cullin domain of anaphase promoting complex), and phytohormone (Histidine kinase, Brassinosteroid insensitive shaggy like protein kinase) was found in the interactome. However, indirect association was found among ABC transporters and chromatin remodeling proteins. Further, E2F transcription factor was directly interacting with Retinoblastoma related protein (RBR) of cell cycle regulator and indirectly with SKP1. Response to humidity genes (copines) were found to be directly interacting with auxin efflux carrier and indirectly with ABC transporters. Additionally, genes like calmodulin, aquaporin, ABP1, GID, SCARECROW, ARF, CLAVATA, GRF, WUSCHEL, Expansin, Sucrose synthase were also found in the network and thus, might be involved during various aspects of growth. Overall, based on PPI interactome analysis, 47 key genes identified as major hubs, interacting with more than 2679 proteins, were important candidates to regulate growth in bamboo.

**Figure 9:**
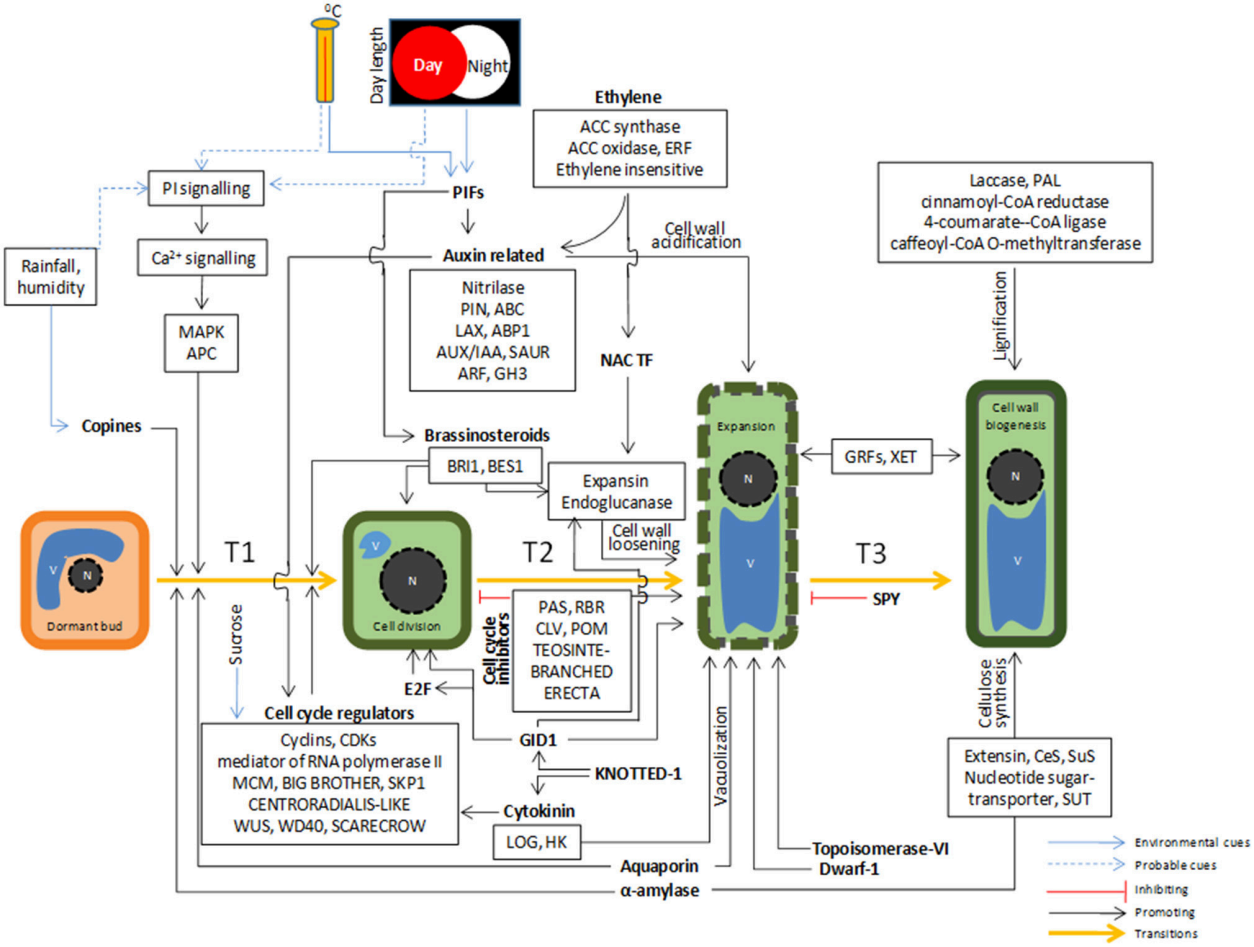
**A hypothesis of complex cross-talk between environmental and cellular signaling cues leading to initiation and persistence of growth in bamboo**.

Growth is a continuous process that occurs throughout the lifespan of a plant (Novikova et al., 2013). In perennial plants like bamboos, annual growth, and dormancy cycles are tightly controlled by interactions between environmental cues and internal factors which help in plant survival during unfavorable conditions (Dennis, 1994). Plants are highly responsive to the change in day length, temperature, and season, modulating their internal gene expression machinery to grow, survive, and reproduce. We hypothesized the existence of a complex and coordinated regulation of various signaling pathways, phytohormones, transcription factors, epigenetic regulators, cell cycle controllers, biosynthetic genes that are possibly involved in initiation and persistence of growth through cell division, cell expansion, and cell wall biogenesis, eventually leading to overall increase in size of bamboo (Figure 9).

It is observed that bamboos undergo vegetative dormancy during winters. This is a protective mechanism in plants to survive and show superior growth during favorable environment. In subtropical bamboos like *Dh*, rhizome arrest cell cycle before the arrival of winters to protect tender young growing tissues. Enormous studies have been done to understand seed dormancy in which ABA plays an important role. However, rhizome dormancy is not well studied at least in case of bamboos at molecular level. In bamboos, ecodormancy is observed, as normal vegetative growth resumes with the arrival of favorable environment (Lang, 1987). New shoot emerges and attains full height only during 3–4 months of monsoon (favorable season); after which the shoot elongation is ceased. Sensing the environmental stimulus is prerequisite to initiate any cellular response. Compared to temperate bamboos, subtropical bamboo (*Dh*) has distinct environmental preferences. Through various mechanisms (discussed below), meristem cells of dormant mother rhizome perceive high humidity, soil water, reducing day length, warm temperature, and sufficient stored energy to initiate growth with the onset of monsoon. Circadian rhythm coordinates internal physiological processes in responses to changing season. Cullin1, a core component of SCF (Skp/Cullin/F-box) complex, plays a key role in diverse developmental processes including embryogenesis, flower development, response to light, and phytohormones (Harmon et al., 2008) was found up-regulated in growing tissues, may possibly be involved in light perception switching the circadian clock for bud break and growth initiation. As evident from differential expression analysis (T1), during monsoon, phytochrome interacting factors (PIFs) integrate light and temperature signals influencing auxin and brassinosteroids level leading to initiation of growth, cell division, subsequent expansion, and vascular differentiation (in growing tissues; Nozue et al., 2007; Rohde and Bhalerao, 2007; Koini et al., 2009; Leivar and Quail, 2011; Oh et al., 2012). Inactivation of specific phytochromes in shades stabilizes various PIFs to promote rapid shoot elongation as observed in Arabidopsis (Wang and Wang, 2015). We found three transcripts of PIF3 supporting growth initiation. Role of Phosphatidylinositol (PI) signaling in growth of temperate bamboo and maize have been reported (Zheng et al., 2010; He et al., 2013). PI signaling activates intracellular calcium signaling pathway in response to changing environment (light, heat, cold, and drought) by rapid change in cytosolic Ca^2+^ ions which acts as an important secondary messenger triggering major physiological changes in response to environmental (like hormones, light, gravity etc.) and developmental signals (Snedden and Fromm, 1998). Calmodulin, the calcium binding proteins transduce Ca^2+^ signals to wide variety of biochemical changes leading to cell division (Snedden and Fromm, 1998, 2001; Yang and Poovaiah, 2003. In *Dh*, three genes of PI signaling pathway and copines (Ca^2+^ binding proteins) which respond to humidity promoting cell growth were found up-regulated in growing shoot and rhizome (Yang et al., 2006). Under permissible growth conditions, gibberellin induced beta-glucosidase and alpha-amylase, which remains repressed during dormancy activates to initiate cell elongation by providing enhanced metabolic flux for bud burst as reported earlier in sessile oak (Derory et al., 2006). In growth season, bamboo already has sufficient stored energy (starch) in rhizome to support rapid growth of new shoots. Cells sense sucrose flux as a primary signal for growth (Ballard and Wildman, 1964; Koch, 1996) in order to commence proliferation by activating cyclin D genes as observed from up-regulated cyclins in our data. Thus, plant cyclin D family appears to be crucial for determining cell's entry into the cell cycle (Koch, 1996; Roitsch, 1999).

To attain characteristic size and shape, molecular processes involved in growth needs to be tightly coordinated through cell proliferation and subsequent cell expansion (Piazza et al., 2005; Tsukaya, 2006; Rodriguez et al., 2010). Cell proliferation occurs in the specialized regions called meristems, a rich source of mitogenic components (such as phytohormones and carbohydrates) that regulate cell cycle progression. Role of transcription factors and hormones in meristem maintenance and cell expansion have been reported in many studies (Gutierrez, 2005; Hay and Tsiantis, 2005; Piazza et al., 2005; De Veylder et al., 2007; Wang et al., 2009; Rodriguez et al., 2010; Peng et al., 2013a). We found seven transcription factor families, namely GRAS (18 transcripts), NAC (11), WUSCHEL (1), KNOTTED-1-like homeobox (2), GRF (17), E2F (1), ARF (49), and WD (130) playing significant role in meristem maintenance. KNOTTED-like homeobox proteins are essential TF in maintaining meristem function by regulating cytokinin and gibberellin (GA) biosynthesis (Piazza et al., 2005). GA promotes cell cycle by inhibiting cell cycle inhibitors and promoting E2F expression (Magyar et al., 2005; Sablowski and Carnier Dornelas, 2014). Auxins and cytokinins are the most extensively studied phytohormones that act as positive intracellular signals regulating cell cycle, cell elongation, cell differentiation, and cell wall relaxation directly or indirectly by activating cyclins and CDKs (Cho and Wang, 2010; Hayashi, 2012; El-Showk et al., 2013; Novikova et al., 2013; Sablowski and Carnier Dornelas, 2014). In our data expression profiles of genes involved in auxin biosynthesis, binding, and transport were up-regulated in growing tissues in T1 and T3 transitions. Two auxin responsive gene families, namely Aux/IAA and SAUR were found to be differentially expressed during both the transitions. Cytokinin and sucrose can activate the transcription of cyclin D, which activates CDKs to mark cell's entry into the cell cycle (Doonan, 2000), thus playing dual role in dormant bud break and cell proliferation. Cytokinins are involved in diverse physiological processes like meristem maintenance, root branching, leaf senescence, inflorescence development, stress tolerance, shoot growth, and seed germination (Müller and Sheen, 2007). Expression of histidine kinase (cytokinin receptor) and LOG (cytokinin biosynthesis) correlated with growth of bamboo. Ethylene regulates auxin biosynthesis and leads to cell division and cell elongation (Novikova et al., 2013). Auxin binding protein 1 (ABP1) was found to mediate auxin-induced cell division and cell elongation (Chen et al., 2001). Ethylene, auxin, cytokinin, brassinosteroids, gibberellin related genes were up-regulated in growing tissues facilitating cell division (either directly or indirectly through cell cycle regulators). Phytohormone mediated cell wall acidification together with aquaporin, endoglucanase, XETs, and expansins facilitates cell expansion (Kretzschmar et al., 2011; Hayashi, 2012; Péret et al., 2012; Wolf et al., 2012; Pei et al., 2013; Sablowski and Carnier Dornelas, 2014). Expression profiles of most of the genes involved in maintenance of cell growth were broadly in accordance of previous report in temperate bamboo (He et al., 2013; Peng et al., 2013a). Several cell division inhibiting genes like PAS, RBR, CLAVATA, POM1 (Harrar et al., 2003; Gutierrez, 2005; Wang et al., 2009) were up-regulated in growing stages. Based on our sampling, growing tissues consists of dividing and elongating cells as well. Hence, it is reasonable to get genes involved in cell cycle progression as well as inhibition to be expressed in GR and GS. Often, elongating cells undergo endoreduplication altering DNA to cytoplasmic ratio resetting cell size threshold (Mizukami, 2001). We found up-regulation of DNA topoisomerase VI genes in growing tissues, specifically involved in removing DNA entanglements during endocycle (Sugimoto-Shirasu and Roberts, 2003). Futuristically, it would be interesting to carry out detailed cytology to better understand this process in bamboos.

For providing mechanical strength to newly divided and elongated cell wall, synthesis of new cell wall components is essential. Plant growth and development rely mainly upon the partitioning of assimilated photosynthates between sources and sink tissues (Baldet et al., 2006). Cell wall is a major sink organ for storage of assimilated photosynthates as cellulose (major structural polymer). Cellulose synthesis is tightly regulated by coordinated activity of sucrose synthase and cellulose synthase (Babb and Haigler, 2001). Expression profiles of starch and sucrose metabolism genes, and transport correlated with cell wall biosynthesis genes facilitating C-fixation in developing shoot. We found coordinated expression of cellulose synthase, sucrose synthase, XETs, extensins, alpha amylase, and sugar transporters to translocate stored nutrients from mother rhizome to growing cell wall. Lignin biosynthesis genes were also found to be expressed contributing to wood formation as previously reported in *P. edulis* (Peng et al., 2013a).

Expression profiles of various epigenetic regulators including DNA methyltransferases, histone acetylases, and deacetylases, chromatin remodeling proteins were found to play significant role in regulating expression of several other genes determining vegetative growth and dormancy cycle as previously seen in chestnut (Santamaría et al., 2009). However, extensive work needs to be done to better understand the role of epigenetic modifiers in bamboo growth.

## Author contributions

AB, RKS: Conceived and designed the experiments; AB, RS: performed the experiments; AB, RS, GS, PS: Analyzed the data; AB, RKS: wrote the paper; RKS: editing and approval of final version of manuscript.

### Conflict of interest statement

The authors declare that the research was conducted in the absence of any commercial or financial relationships that could be construed as a potential conflict of interest.
