# Supplementary material for: Genome-Wide Transcriptional Profiling to Elucidate Key Candidates Involved in Bud Burst and Rattling Growth in a Subtropical Bamboo (Dendrocalamus hamiltonii)
Source: Front Plant Sci. 2017 Jan 11;7:2038. doi: 10.3389/fpls.2016.02038 (PMC5225089; doi:10.3389/fpls.2016.02038)
Supplement: Supplementary file 1 [file Table1.DOCX]

**Supplementary Table S1** List of expression primer sets of selected transcripts used for Real time-PCR analysis.

| **S. No.** | **Gene code** | **Gene** | **Transcript ID** | **Expression primer sequences (5’🡪3’)** | **T_m_°C** |
| --- | --- | --- | --- | --- | --- |
| **1** | *SUS* | Sucrose synthase | DH_4415 | Fwd: TGCCAGTTCACAGCAGACCTT  Rev: CGGTAGAGCCCAGGAAGAGTG | 63.30 |
| **2** | *CALM* | Calmodulin | DH_8297 | Fwd: GCTCCCATTCTTGGACAGACC  Rev: CATTGTTTATTCGGCGTGATGA | 62.95 |
| **3** | *CES* | Cellulose synthase A | DH_18332 | Fwd: GCATACCTAAACGGGCTGCAT  Rev: GCCCACCACCATATCCATACC | 63.34 |
| **4** | *GRAS* | GRAS Family TF (Scarecrow like) | DH_2271 | Fwd: TGTGCTAGAGCGGTGGAAGAG  Rev: CACCATGTAGGCTCCCAACCT | 62.93 |
| **5** | *AEC* | Auxin Efflux Carrier | DH_31583 | Fwd: AAGTGGTGGCGCATCTTCTC  Rev: TCATGGTGTAGGGGTTGTTGG | 62.68 |
| **6** | *EXP* | Expansin B-2 | DH_32525 | Fwd: GACGACCGCAACGACGAG  Rev: ACGAGCACCGCCAGATACAC | 63.55 |
| **7** | *PIP4K* | Phosphatidylinositol 4-kinase | DH_16172 | Fwd: GGGTGCTGGTGCTCTCAACTA  Rev: TCACTTGGCTCCTCTTCTTTCC | 62.10 |
| **8** | *WD40* | WD-40 repeat | DH_22378 | Fwd: GCGGATGGCACAGAGGAC  Rev: TGCGGGCTCAAGTGGTCTA | 62.90 |
| **9** | *CDK* | Cyclin dependent kinase C | DH_3960 | Fwd: CTTGCTGATTTTGGCCTTGC  Rev: ACCATACCTTGTGCTTCCGAGT | 62.15 |
| **10** | *CLV1* | CLAVATA1 | DH_33635 | Fwd: CTCATTGTCCTCGGCTTGCTT  Rev: CCTCGCTGGCTTTCTTCAGTG | 63.93 |
| **11** | *ABC* | ABC transporter | DH_18471 | Fwd: GAGGAACCATTGAGGAAAGCAA  Rev: TCCCACCATACCAGAAGTCCA | 62.63 |
| **12** | *ARF* | Auxin response factor | DH_13302 | Fwd: CTGTAAGCCCTCCTGTCAACC  Rev: TGGAAGACCATTTTGCTGTGA | 61.05 |
| **13** | *CYP*  *(HK)* | Cyclophilin 5 (Housekeeping) | DH_15018 | Fwd: ACGGGAAGCATGTTGTGTTTG  Rev: AGTTCTCCGCTGTCTGCAATG | 62.82 |

Tm°C: melting temperature

**Quantitative real time-PCR conditions:**

The PCR amplification program was as follows: 95°C for 10 min; 35 cycles of 95°C for 30s, Tm°C for 30 sec and 72°C for 30s; followed by a melting-curve program of 95°C for 1min, 55°C for 30s and 95°C for 30s.
