# Supplementary material for: Genome-Wide Transcriptional Profiling to Elucidate Key Candidates Involved in Bud Burst and Rattling Growth in a Subtropical Bamboo (Dendrocalamus hamiltonii)
Source: Front Plant Sci. 2017 Jan 11;7:2038. doi: 10.3389/fpls.2016.02038 (PMC5225089; doi:10.3389/fpls.2016.02038)
Supplement: Supplementary file 2 [file Table2.DOCX]

Supplemenatary Table S2 Correlation between *D. hamiltonii* growth rate and environmental factors.

| **Growth rate vs Factor** | **Correlation**  **coefficient *(r)*** | **Significance**  **(*P-value*)** |  |
| --- | --- | --- | --- |
| **Temperature** | 0.61152806 | **0.02013** |  |
| **Humidity** | 0.80616928 | 0.00049 |  |
| **Day length** | 0.78049899 | 0.00099 |  |
