# Supplementary material for: Genome-Wide Transcriptional Profiling to Elucidate Key Candidates Involved in Bud Burst and Rattling Growth in a Subtropical Bamboo (Dendrocalamus hamiltonii)
Source: Front Plant Sci. 2017 Jan 11;7:2038. doi: 10.3389/fpls.2016.02038 (PMC5225089; doi:10.3389/fpls.2016.02038)
Supplement: Supplementary file 3 [file Table3.DOCX]

**Supplementary Table S3** Statistics of *D. hamiltonii* transcriptome assembly

| **Total reads** | **66,465,634** |
| --- | --- |
| High quality reads | 56,907,264 |
| High quality bases sequenced | 4,077,122,268 |
| No. of assembled transcripts | 39,603 |
| Size of assembled transcripts (Mb) | 21.9 |
| Maximum transcript length (bp) | 5,038 |
| Average transcript length (bp) | 553 |
| N50 length (bp) | 559 |
| N75 length (bp) | 408 |
| GC content | 47.6 % |
