# Supplementary material for: Genome-Wide Transcriptional Profiling to Elucidate Key Candidates Involved in Bud Burst and Rattling Growth in a Subtropical Bamboo (Dendrocalamus hamiltonii)
Source: Front Plant Sci. 2017 Jan 11;7:2038. doi: 10.3389/fpls.2016.02038 (PMC5225089; doi:10.3389/fpls.2016.02038)
Supplement: Supplementary file 7 [file Table7.DOCX]

**Supplementary Table S7** Summarization of protein-protein interaction (PPI) network of key growth related genes identified in *D. hamiltonii* transcriptome analysis with *Arabidopsis* proteome

| **Category** | **Transcript ID** | **Arabidopsis ID** | **No. of nodes** | **Description** |
| --- | --- | --- | --- | --- |
| **Environmental signal perception** | DH_642 | AT1G26830 | 58 | Cullin 3 |
|  | DH_567 | AT4G02570 | 91 | Cullin 1 |
|  | DH_1129 | AT2G04660 | 42 | Cullin |
|  | DH_20675 | AT5G58690 | 28 | Phosphatidylinositol-specific phospholipase C5 |
|  | DH_18949 | AT1G06390 | 36 | GSK3/SHAGGY-like protein kinase 1 |
|  | DH_8585 | AT5G26751 | 38 | Shaggy-related kinase 11 |
|  | DH_12355 | AT4G14350 | 65 | AGC (cAMP-dependent, cGMP-dependent and protein kinase C) kinase family protein |
|  | DH_836 | AT4G33080 | 31 | AGC (cAMP-dependent, cGMP-dependent and protein kinase C) kinase family protein |
| **Phytohormones** | DH_2198 | AT2G01830 | 78 | CHASE domain containing histidine kinase protein |
|  | DH_22381 | AT5G35750 | 49 | Histidine kinase 2 |
|  | DH_2984 | AT1G27320 | 90 | Histidine kinase 3 |
|  | DH_7526 | AT2G39480 | 60 | ABC transporter B family |
|  | DH_17587 | AT3G62150 | 43 | ABC transporter B family |
|  | DH_4468 | AT2G47000 | 34 | ATP binding cassette subfamily B4 |
| **Epigenetic modulators** | DH_882 | AT4G38130 | 89 | Histone deacetylase 1 |
|  | DH_7977 | AT3G54610 | 103 | Histone acetyltransferase of the GNAT family 1 |
|  | DH_6711 | AT1G79000 | 35 | Histone acetyltransferase of the CBP family 1 |
|  | DH_20297 | AT3G12980 | 36 | Histone acetyltransferase of the CBP family 5 |
|  | DH_5900 | AT4G26600 | 130 | S-adenosyl-L-methionine-dependent methyltransferases superfamily protein |
|  | DH_5152 | AT2G18760 | 67 | Chromatin remodeling 8 |
|  | DH_731 | AT2G13370 | 75 | Chromatin remodeling 5 |
| **Transcription factors** | DH_9769 | AT2G31300 | 29 | Actin-related protein C1B |
|  | DH_4563 | AT5G13480 | 57 | Transducin/WD40 repeat-like superfamily protein |
|  | DH_4567 | AT4G34460 | 72 | GTP binding protein beta 1 |
|  | DH_286 | AT2G36010 | 32 | E2F transcription factor 3 |
|  | DH_11769 | AT3G10530 | 60 | Transducin/WD40 repeat-like superfamily protein |
|  | DH_627 | AT3G16650 | 40 | Transducin/WD40 repeat-like superfamily protein |
|  | DH_5527 | AT3G44530 | 69 | Homolog of histone chaperone HIRA |
|  | DH_8977 | AT4G00800 | 65 | Transducin family protein / WD-40 repeat family protein |
|  | DH_3983 | AT4G18905 | 38 | Transducin/WD40 repeat-like superfamily protein |
|  | DH_2644 | AT3G18860 | 130 | Transducin family protein / WD-40 repeat family protein |
|  | DH_14756 | AT5G15550 | 73 | Transducin/WD40 repeat-like superfamily protein |
|  | DH_16243 | AT2G40360 | 85 | Transducin/WD40 repeat-like superfamily protein |
|  | DH_7595 | AT3G62770 | 124 | Transducin/WD40 repeat-like superfamily protein |
|  | DH_8258 | AT5G54200 | 100 | Transducin/WD40 repeat-like superfamily protein |
|  | DH_11572 | AT1G15440 | 211 | Periodic tryptophan protein 2 |
|  | DH_417 | AT4G04940 | 34 | Transducin family protein / WD-40 repeat family protein |
|  | DH_3056 | AT4G07410 | 38 | Transducin family protein / WD-40 repeat family protein |
|  | DH_2553 | AT1G49540 | 72 | WD family, elongator protein 2 |
| **Cell cycle regulators** | DH_18306 | AT4G34210 | 51 | SKP1-like 11 |
|  | DH_6989 | AT3G12280 | 285 | Retinoblastoma-related 1 |
|  | DH_4078 | AT5G02500 | 44 | Heat shock cognate protein 70-1 |
|  | DH_29618 | AT5G06150 | 115 | Cyclin family protein |
|  | DH_10301 | AT5G45190 | 57 | Cyclin family protein |
|  | DH_33205 | AT5G46280 | 50 | Minichromosome maintenance (MCM2/3/5) family protein |
|  | DH_3093 | AT1G15780 | 65 | Mediator of RNA pol II |
| **Cell wall biogenesis** | DH_30703 | AT1G02730 | 48 | cellulose synthase-like D5 |
