# Supplementary material for: Genome-Wide Transcriptional Profiling to Elucidate Key Candidates Involved in Bud Burst and Rattling Growth in a Subtropical Bamboo (Dendrocalamus hamiltonii)
Source: Front Plant Sci. 2017 Jan 11;7:2038. doi: 10.3389/fpls.2016.02038 (PMC5225089; doi:10.3389/fpls.2016.02038)
Supplement: Supplementary file 8 [file Presentation1.PDF]

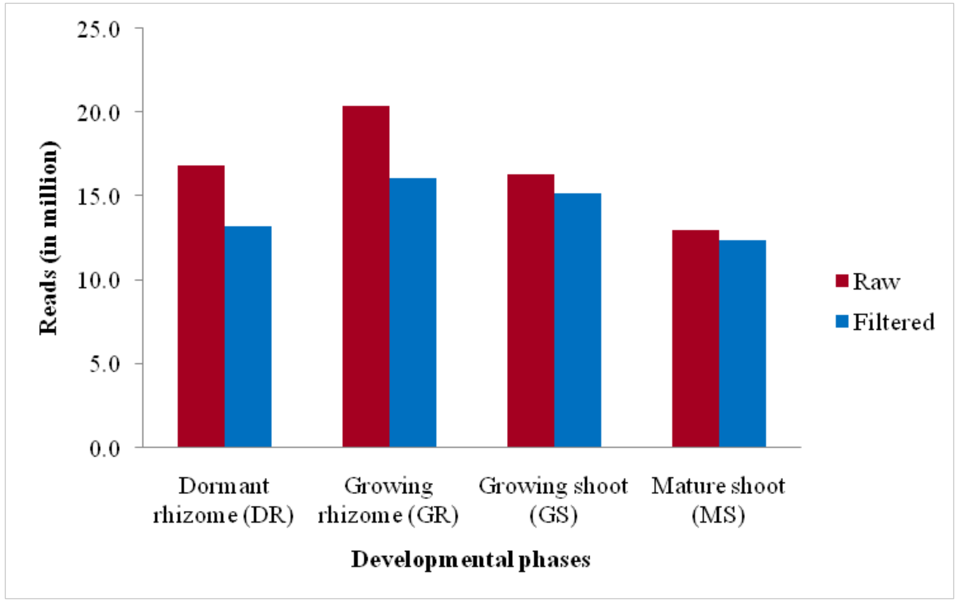


**Fig. S1** Summary of sequence reads generated from transcriptome sequencing of different developmental phases of shoot and rhizome.

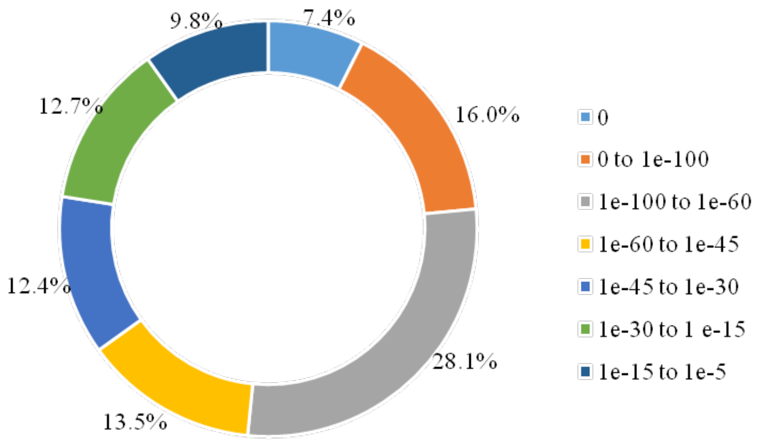


**Fig. S2** Transcript annotation showing E-value distribution of hits with NR database.

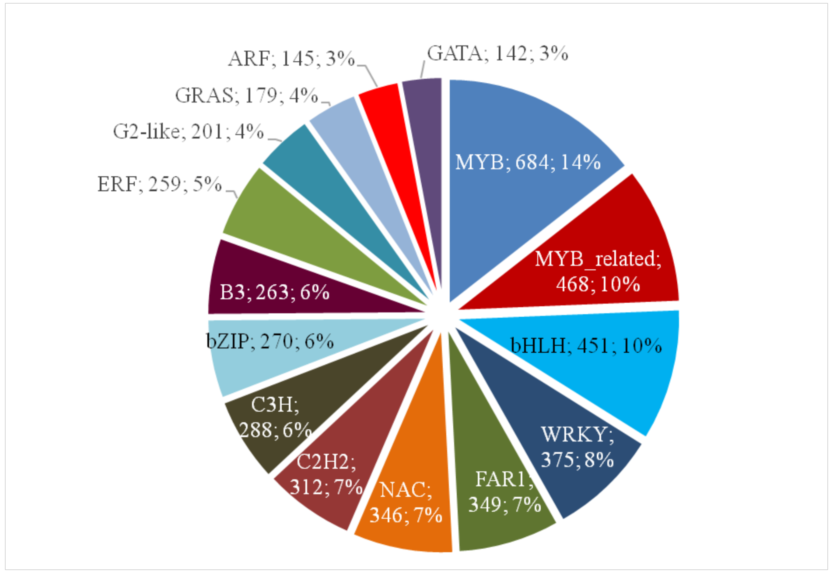


**Fig. S3** Occurrence of top 15 transcription factor families

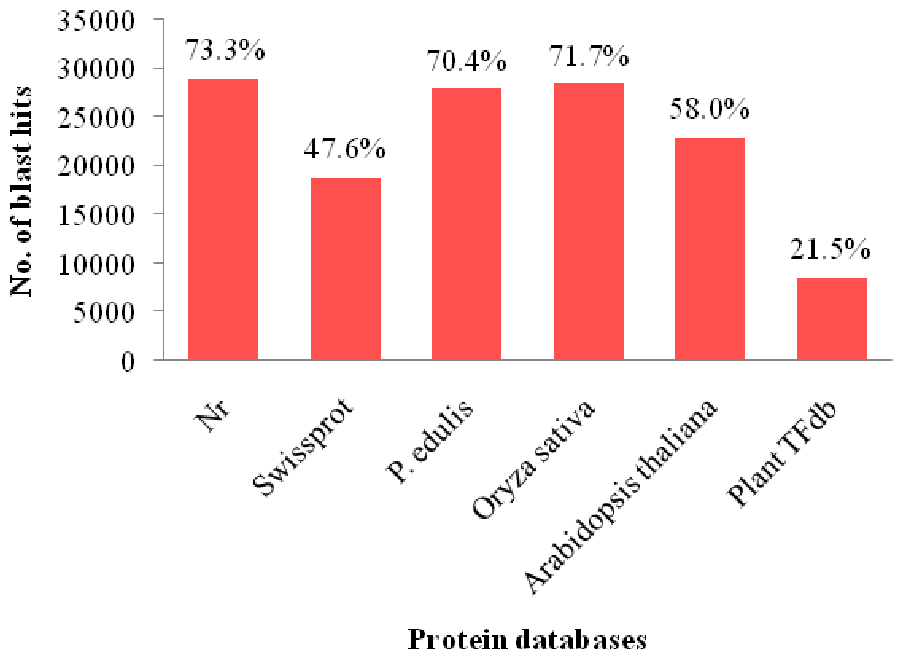


**Fig. S4 Summary of sequence annotations against various protein databases.**

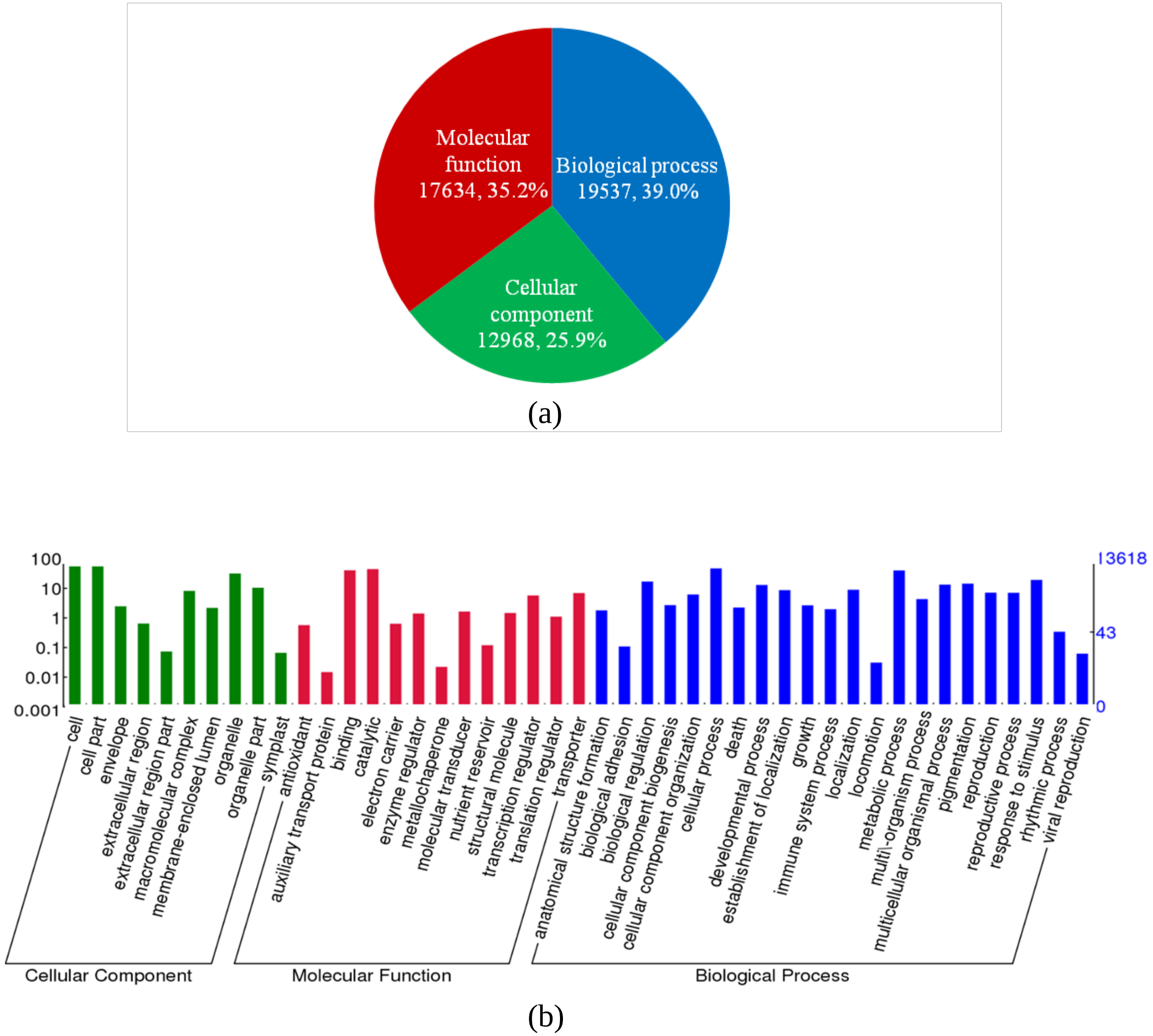


**Fig. S5**. Gene ontology classification of *D. hamiltonii* transcripts. (a) Major GO categories: biological processes, molecular function and cellular components and (b) 45 sub categories under GO classification.

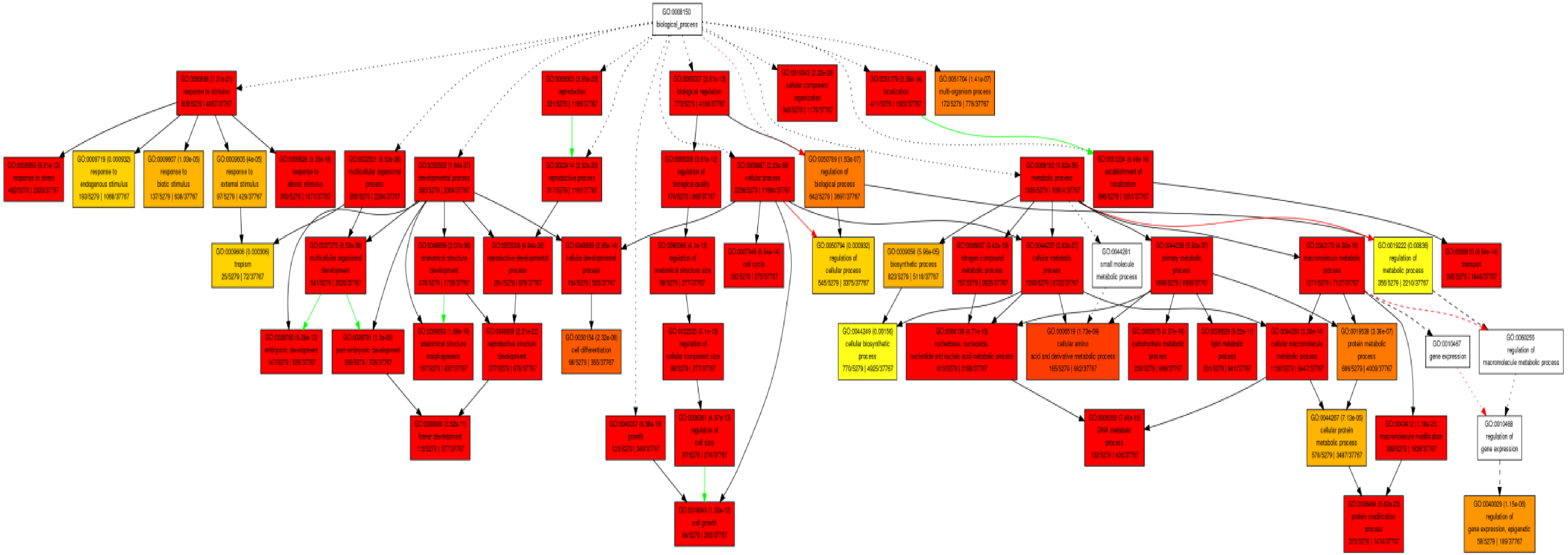


**Fig. S6 Gene ontology enrichment analyses of differentially expressed transcripts in growing shoot showing enriched biological processes.**

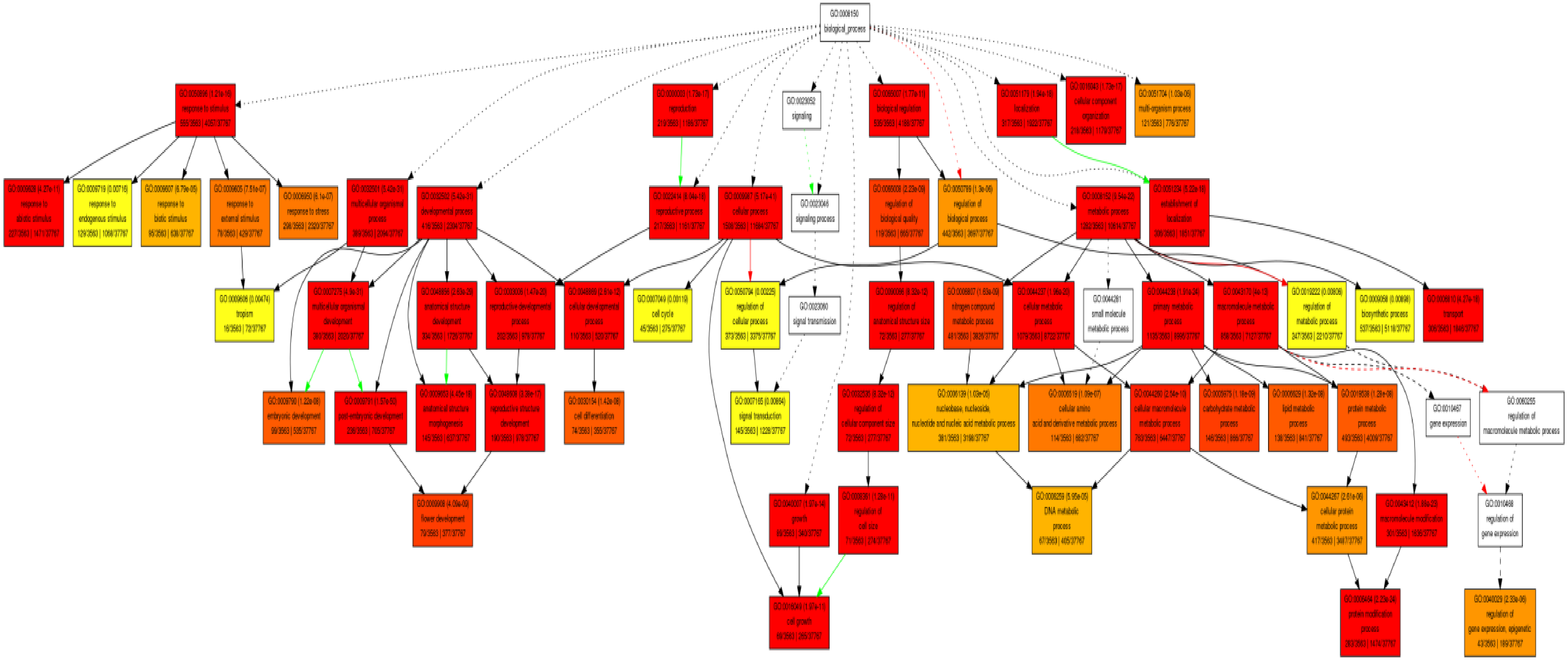

**Fig. S7** Gene ontology enrichment analyses of differentially expressed transcripts in growing rhizome showing enriched biological processes.

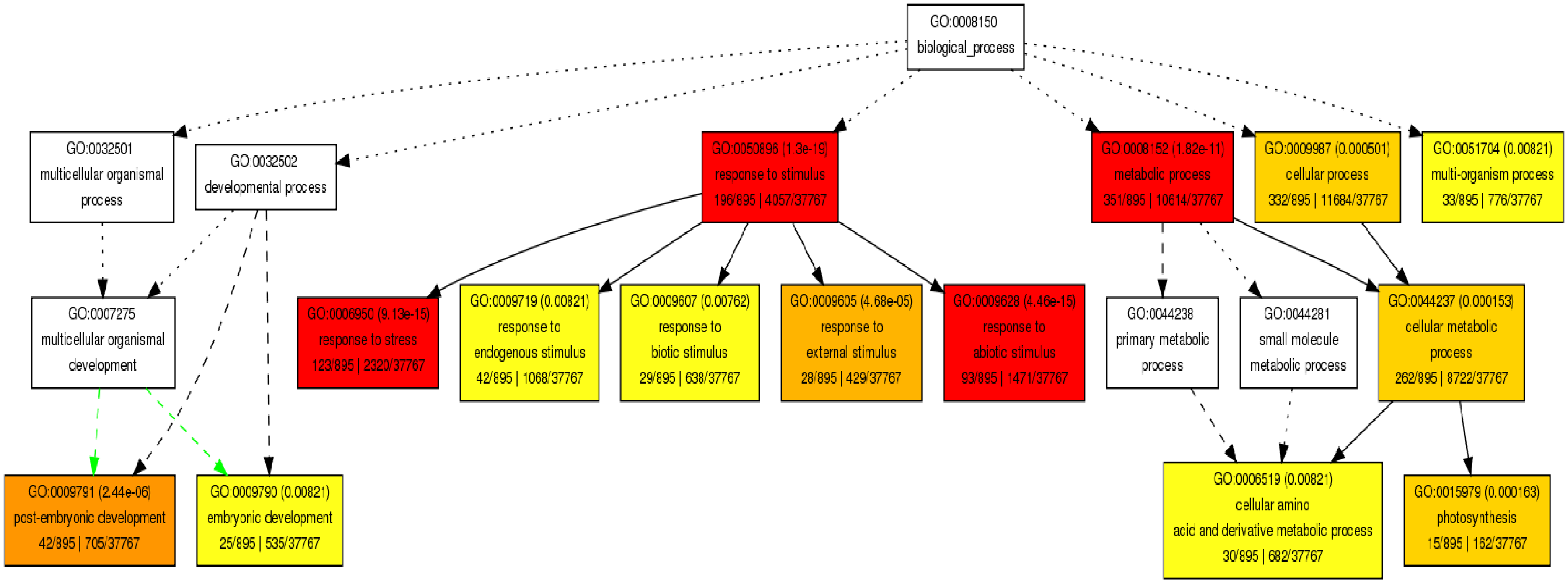


**Fig. S8** Gene ontology enrichment analyses of differentially expressed transcripts in dormant rhizome showing enriched biological processes.

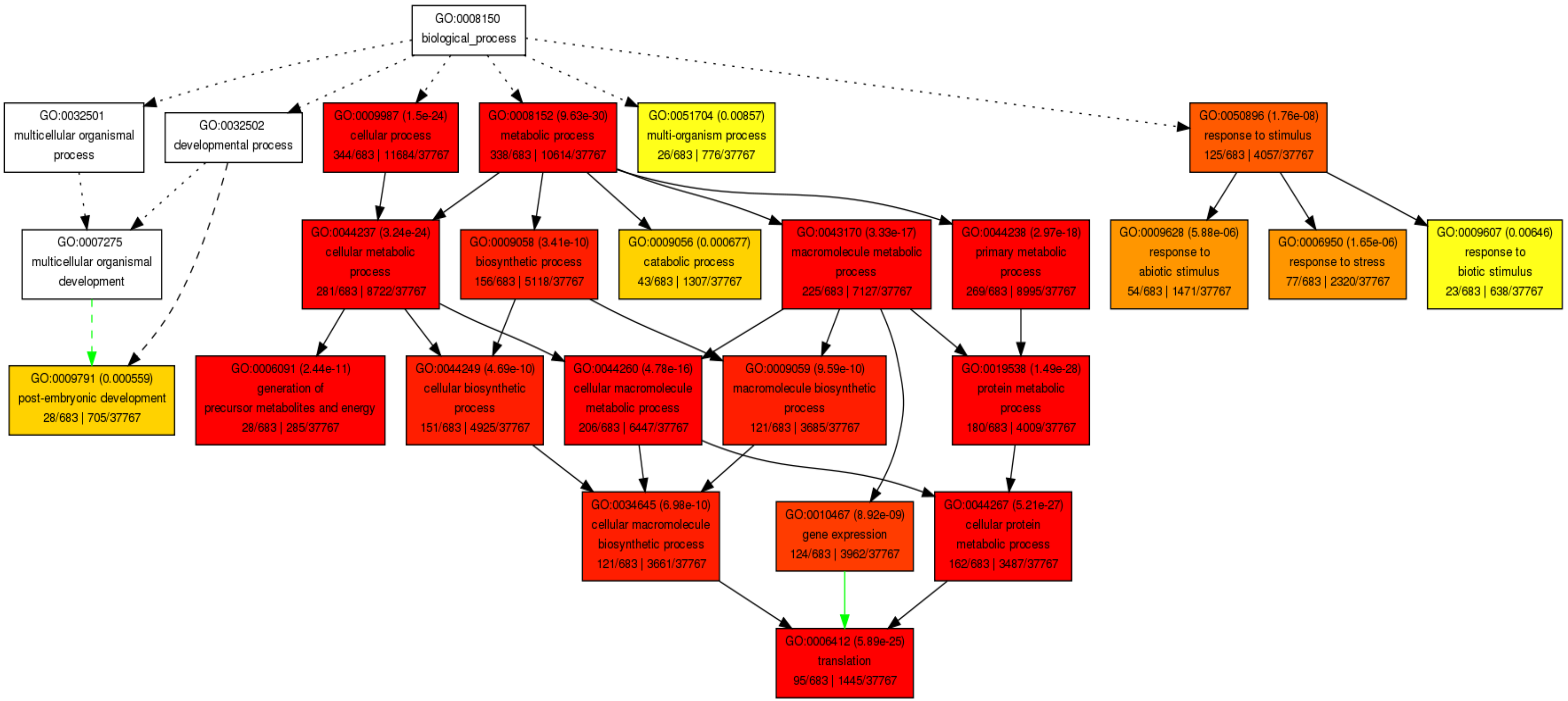


**Fig. S9** Gene ontology enrichment analyses of differentially expressed transcripts in mature shoot showing enriched biological processes.
